# Supplementary material for: IMMUNE AND MOLECULAR CORRELATES OF RESPONSE TO IMMUNOTHERAPY REVEALED BY BRAIN-METASTATIC MELANOMA MODELS
Source: bioRxiv. 2024 Sep 25:2024.08.26.609785. Preprint. [Version 2] doi: 10.1101/2024.08.26.609785 (PMC11451731; doi:10.1101/2024.08.26.609785)
Supplement: Supplementary file 1 [file NIHPP2024.08.26.609785v2-supplement-1.pdf]

Daugherty-Lopès et al. FigS1

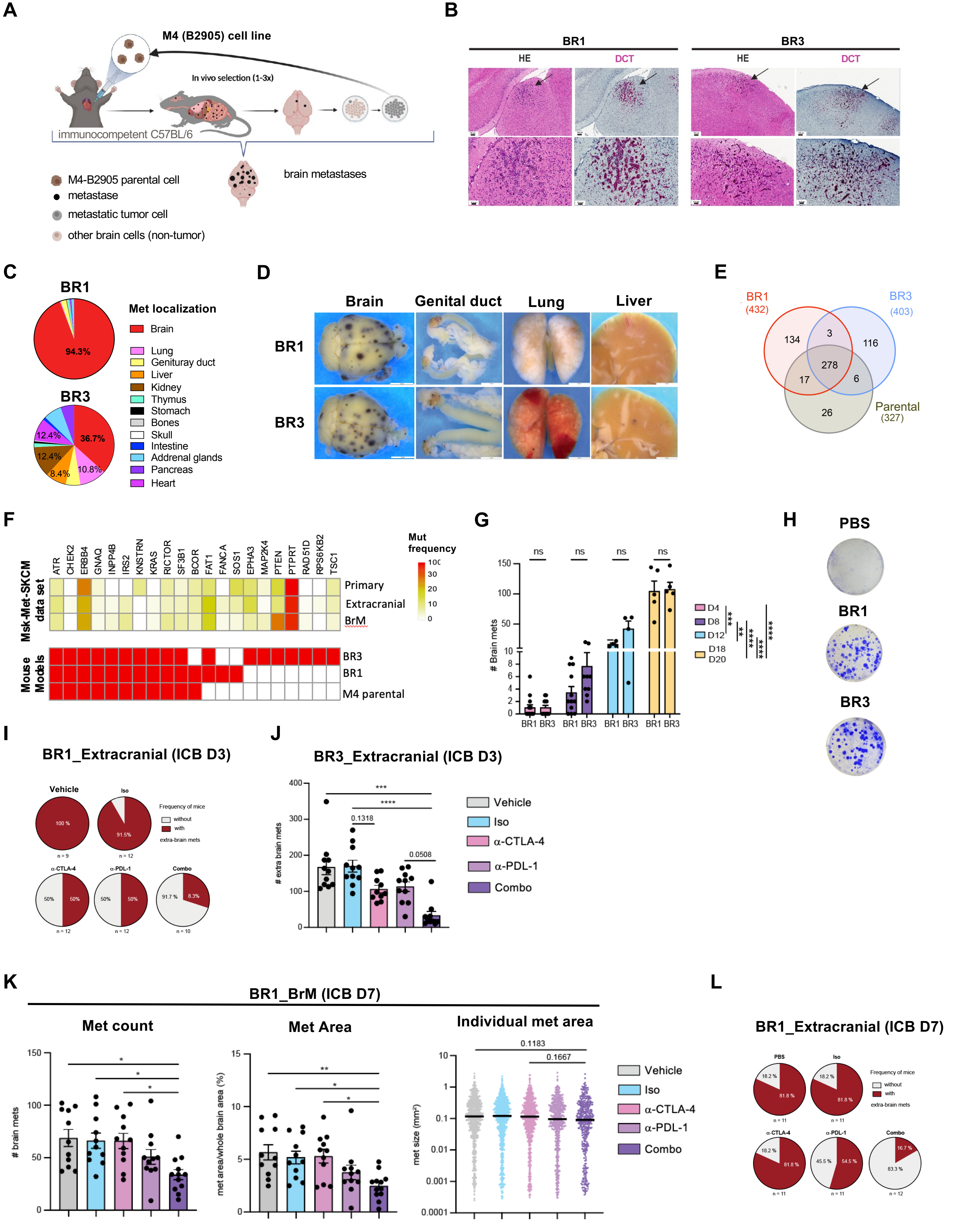

Figure S1. Characterization of the metastatic potential, mutational landscapes, and ICB responses of BR1 and BR3 models, related to Figure 1.

(A) Experimental design for the development of BrM melanoma models. (B) Hematoxylin / eosin (HE) and dopachrome tautomerase (DCT; pink) immunostaining images of brain sagittal sections showing BR1 (left panels) and BR3 (right panels) BrMs. Magnification 15x (top) and 40x (bottom). (C) Percentage of metastases per organ in BR1 (top) and BR3 (bottom) models. (D) Representative stereomicroscope images of the indicated organs from BR1 and BR3 BrM-bearing animals. (E) Venn diagram showing the number of genes mutated and their overlap between the indicated cell lines. (F) Mutation frequency of indicated genes found in the brain-metastatic cell lines (marked in red in bottom heatmap), BrMs, extracranial metastases, and primary tumors from patients with BrMs from the MSK MetTropism cutaneous melanoma (SKCM) dataset. Color scale represents the mutation frequency in the indicated sample group. (G) BrM counts at the indicated time point after intracardiac injection (i.c.) in BR1 and BR3 bearing animals. D18/D20 indicate harvest at experimental endpoint. (H) Macroscopic pictures of cultured cell colonies recovered from mouse brains from BR1 and BR3 bearing animals or non-BrM control (PBS). (I) Proportion of mice with or without BR1 extracranial metastases after treatment on day 3 post tumor injection with PBS (vehicle), isotype control (Iso), anti-CTLA-4 (α-CTLA-4) or anti-PD-L1 (α-PD-L1) alone, or anti-PD-L1 + anti-CTLA-4 combination therapy (Combo). (J) BR3 extracranial metastatic count per mouse in animals treated as in I. (K) BR1 BrM count (left), total brain metastatic area per individual brain (middle), and size of individual BrM lesion (right) from animals treated as in I starting treatment on day 7 post tumor injection. (L) Proportion of animals from K with or without extracranial metastases. B one representative of 2 experiments, n=2-5 mouse/group/experiment. C data from 3 experiments combined, n=2-5 mouse/group/experiment. D one representative of 3 independent experiments, n=2-5 mouse/group/experiment. G data combined from 2 independent experiments, n = 2-5 mouse/group/experiment. I-L representative of 2 independent experiments per model, n = 9-12/group/experiment. G, J, K data shown as mean ± SEM *p <0.05, **p <0.01, ***p < 0.001, ****p < 0.00001. See also Table S1

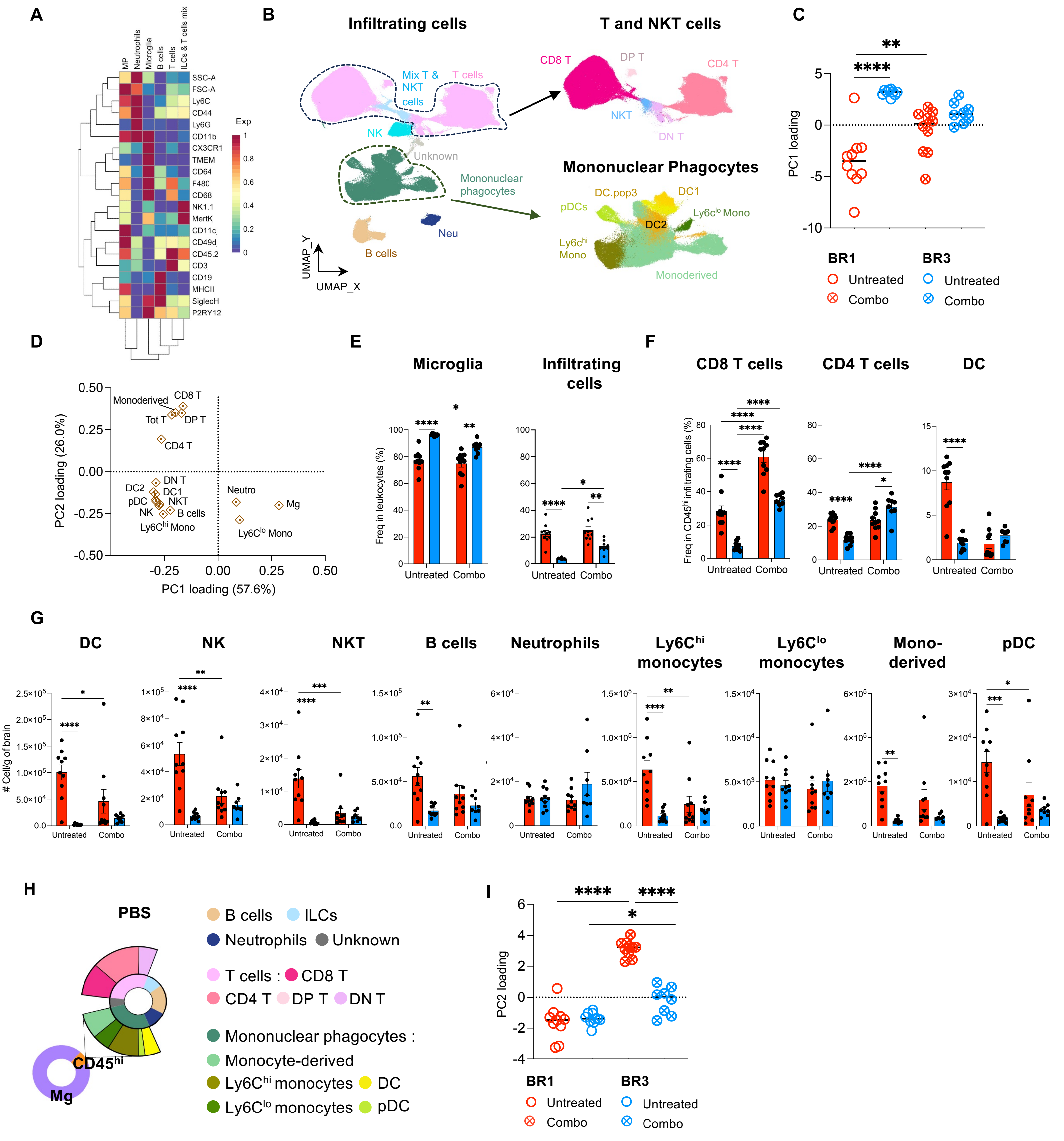
A
B
Infiltrating cells
T and NKT cells
C
Mix T & NKT cells
T cells
NK
Unknown
Mononuclear phagocytes
Neu
B cells
UMAP_Y
UMAP_X
CD8 T
DP T
CD4 T
NKT
DN T
Mononuclear Phagocytes
DC.pop3
DC1
pDCs
Ly6clo Mono
DC2
Ly6chi Mono
Monoderived
PC1 loading
BR1
Untreated
Combo
BR3
Untreated
Combo
D
PC2 loading (26.0%)
PC1 loading (57.6%)
Monoderived
CD8 T
Tot T
DP T
CD4 T
DN T
DC2
DC1
pDC
NKT
NK
B cells
Ly6Chi Mono
Neutro
Mg
Ly6Clo Mono
E
Microglia
Infiltrating cells
Freq in leukocytes (%)
Untreated
Combo
F
CD8 T cells
CD4 T cells
DC
Freq in CD45hi infiltrating cells (%)
G
DC
NK
NKT
B cells
Neutrophils
Ly6Chi monocytes
Ly6Clo monocytes
Mono-derived
pDC
Cell/g of brain
H
PBS
CD45hi
Mg
B cells
ILCs
Neutrophils
Unknown
T cells : CD8 T
CD4 T
DP T
DN T
Mononuclear phagocytes :
Monocyte-derived
Ly6Chi monocytes
DC
Ly6Clo monocytes
pDC
I
PC2 loading
BR1
Untreated
Combo
BR3
Untreated
Combo

Figure S2. Global immune BrTME characterization of untreated and treated BR1 and BR3 bearing brains, related to Figure 2.

(A) Heatmap representing expression level of indicated surface markers (scaled by marker) for each immune cluster with hierarchical clustering indicated on left and markers on top. (B) UMAP projection of indicated immune cells. NKT: natural killer T; T cell subsets: $CD8^+CD4^-$ (CD8 T), $CD8^-CD4^+$ (CD4 T), $CD8^-CD4^-$ (DN T), $CD8^+CD4^+$ (DP T); mononuclear phagocytes including dendritic cells [DC1, DC2, DC.pop3, plasmacytoid DC (pDCs)] and monocytes subsets [$Ly6C^{hi}$ mono and $Ly6C^{lo}$ mono]. (C) Quantification of principal component 1 (PC1) from Figure 2D. (D) PC1 and PC2 loadings from Figure 2D. (E-F) Proportion of indicated immune cells. (G) Absolute number of indicated immune cells per gram of brain. (H) Proportion of microglia (Mg; purple) and infiltrating cells ($CD45^{hi}$; orange) within total leukocytes in control (PBS) brains. Proportion of indicated populations in total infiltrating cells (inset). (I) Quantification PC2 from Figure 2D. C, E-G, and I data shown as mean, n = 8-10/group/experiment. $^*p < 0.05$, $^{**}p < 0.01$, $^{****}p < 0.00001$. See also Table S2.

Daugherty-Lopès et al. FigS3

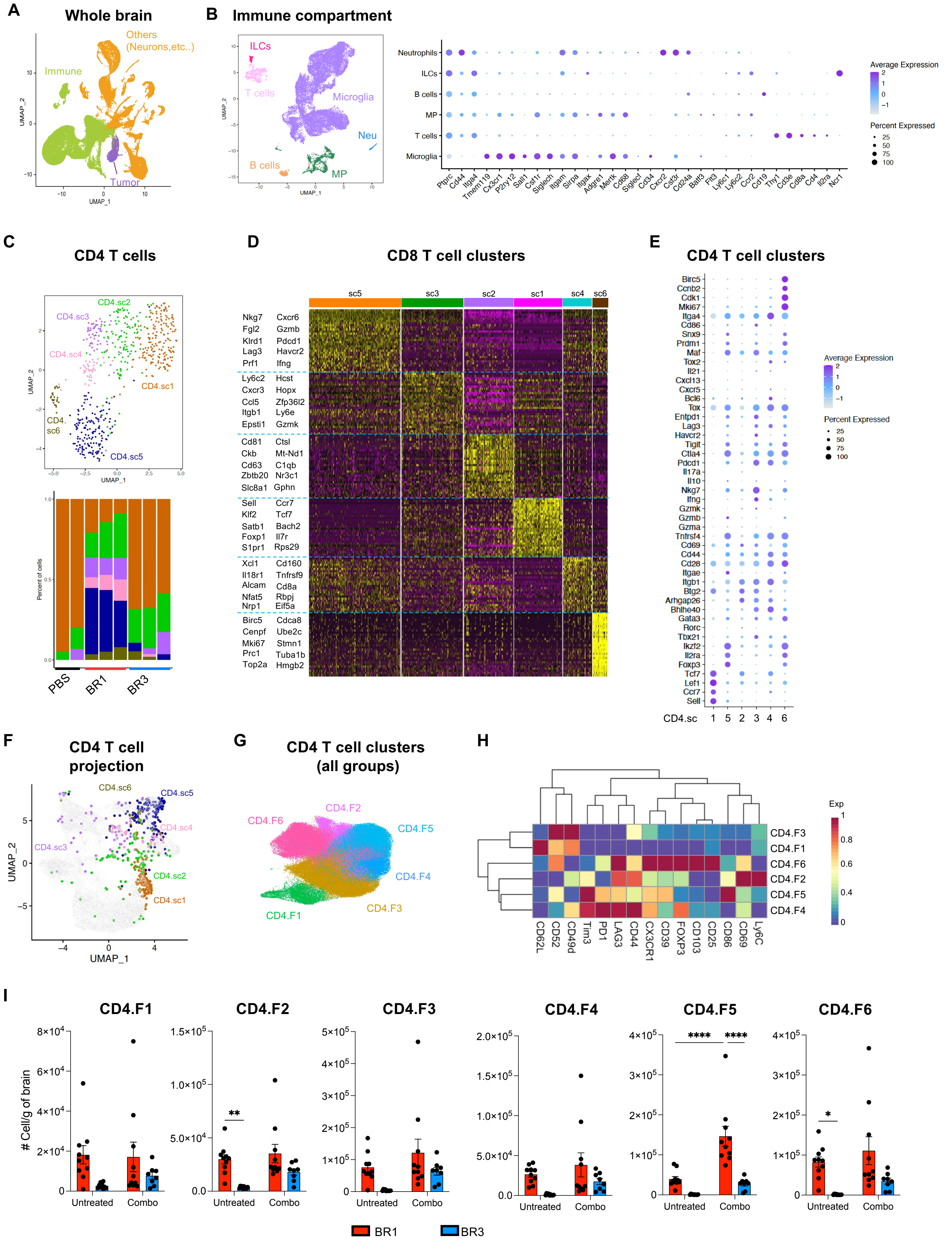

Figure S3. Distinct T cell characteristics found in the ICB-responder model, related to Figure 3.

(A-B) ScRNA-seq analysis of untreated BR1, BR3, and PBS brains. (A) UMAP projection of whole brain cells. (B) UMAP projection of immune cells (left). Dot plot showing the expression of selected genes differentially expressed between clusters (right). (C-F) scRNA-seq analysis of indicated brain T cell populations. (C) UMAP projection of indicated $CD4^+$ T cell clusters among total $CD4^+$ T cells (top). Proportion of indicated $CD4^+$ T cell clusters in total $CD4^+$ T cells per sample (bottom). (D) Heatmap representing expression level of the top 30 $CD8^+$ T cell cluster-specific genes. (E) Dot plot showing the expression of selected genes differentially expressed between $CD4^+$ T cell clusters. (F) Projection of our $CD4^+$ T cell clusters onto the single-cell reference atlas for T cell states ProjecTILs. See figure 4E for the Reference map (left). (G-I) High-parametric flow cytometry analysis of $CD4^+$ T cells from BR1 and BR3 brains untreated or combo treated. (G) UMAP projection of $CD4^+$ T cell populations. (H) Heatmap representing expression level of indicated surface markers (scaled by marker) for each $CD4^+$ T cell cluster with hierarchical clustering indicated on left and markers on top. (I) Absolute number of indicated $CD4^+$ T cell clusters per gram of brain. A-D n = 2-3/group/experiment. F-G Data shown as mean ± SEM, n = 8-10/group/experiment. *p <0.05, ***p < 0.001, ****p < 0.00001. See also Table S3-S4.

Daugherty-Lopès et al. FigS4

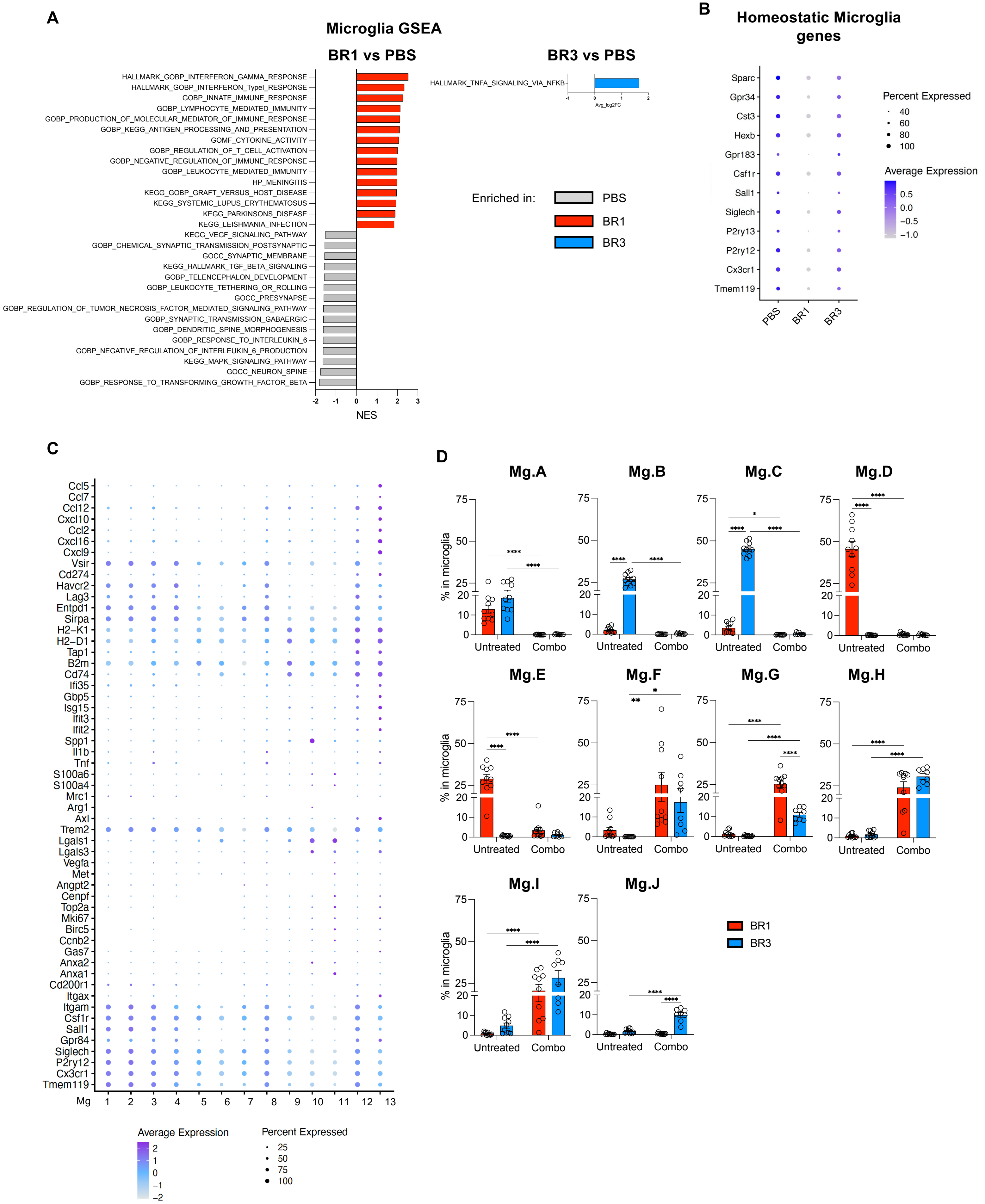

Figure S4. Distinct microglia are found in ICB-responder and resistant BrMs, related to Figure 4.

(A-C) scRNA-seq analysis of microglia from untreated BR1, BR3, and PBS brains. (A) GSEA showing HP, GO, and KEGG pathways selected among the top 10 enriched in total BR1 microglia versus PBS (left) and in total BR3 microglia versus PBS (right) (see Methods). (B) Dot plot showing the expression of homeostatic microglia genes per group. (C) Dot plot showing expression of selected genes differentially expressed between microglia clusters. (D) High-parametric spectral flow cytometry analysis of microglia from BR1 and BR3 bearing animals untreated or combo-treated. Proportion of indicated microglia clusters in total microglia. A-C n = 2-3/group/experiment. D n = 8-10/group/experiment. *$p < 0.05$, **$p < 0.01$, ***$p < 0.001$, ****$p < 0.0000$

Daugherty-Lopès et al. FigS5

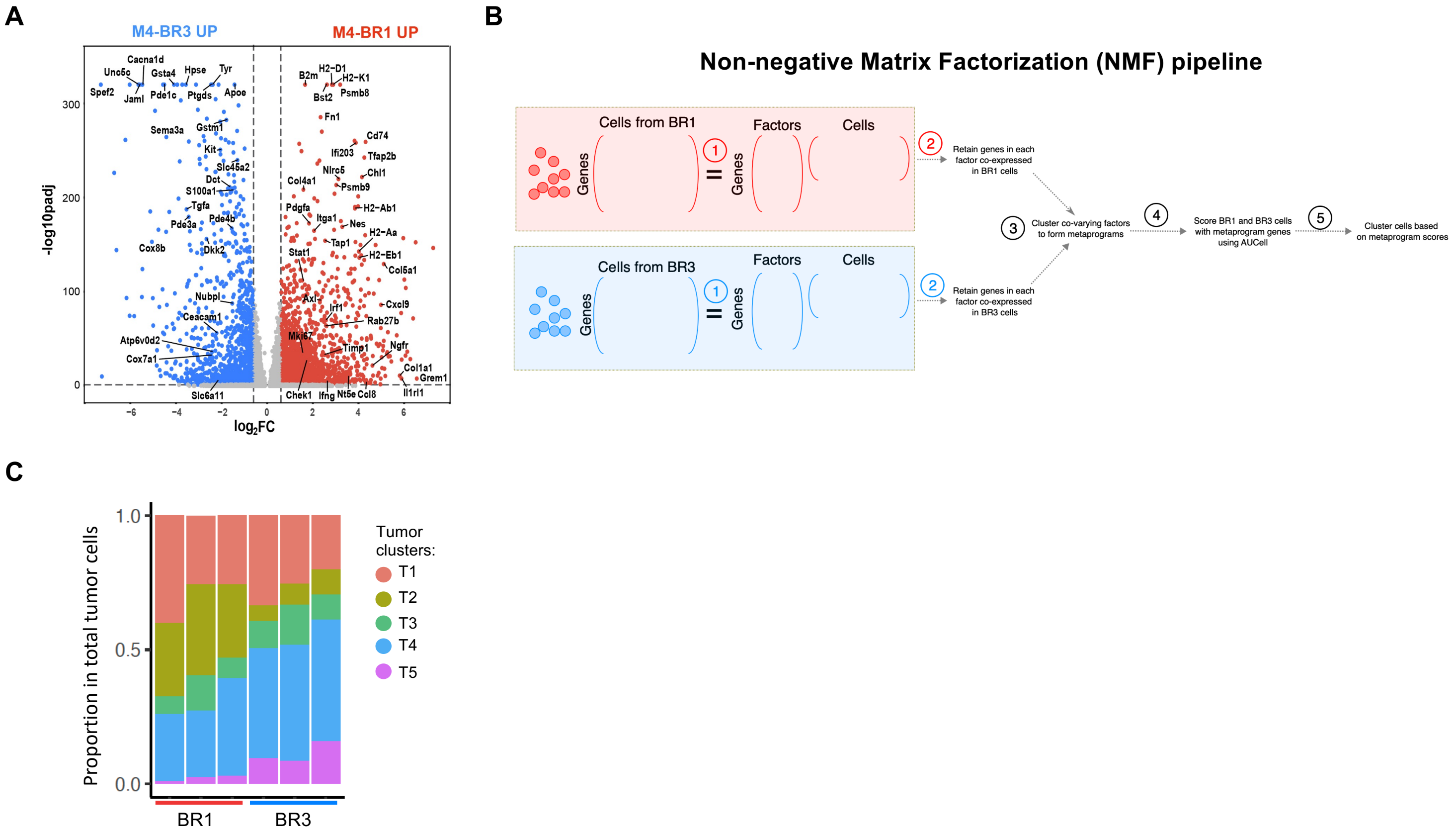

Figure S5. BR1 and BR3 tumor cell molecular characterization by scRNA-seq, related to Figure 5.

scRNA-seq analysis of tumor cells from untreated BR1 and BR3 brains. (A) Volcano plot of BR1 versus BR3 differentially expressed genes. (B) Schematic of metaprogram discovery and their use in clustering malignant cells in BR1 and BR3 models. Step 1: NMF is carried out (k = 30 factors) separately in malignant cells from BR1 and BR3 models based on genes that are expressed in at least 1% of cells in either model. Step 2: Genes with a high weight in each factor are then filtered to retain genes that are co-expressed. Step 3: The activity of each filtered factor is computed across all malignant cells using AUCell. A correlation matrix between all pairs of factors is computed based on these activities, and after hierarchical clustering, correlated factors are grouped together. Genes occurring in at least 25% of constituent factors in a group are retained to form metaprograms. Step 4: AUCell is used to compute the activity of all metaprograms across all malignant cells. Step 5: Cluster cells using Louvain clustering based on metaprogram activity scores. (C) Proportion per sample of indicated tumor clusters. A-C n = 2-3/group. See also Table S5.

A

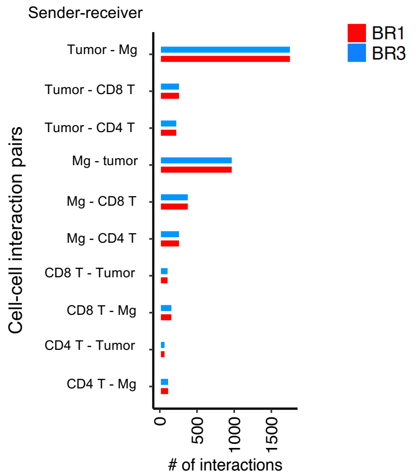

B

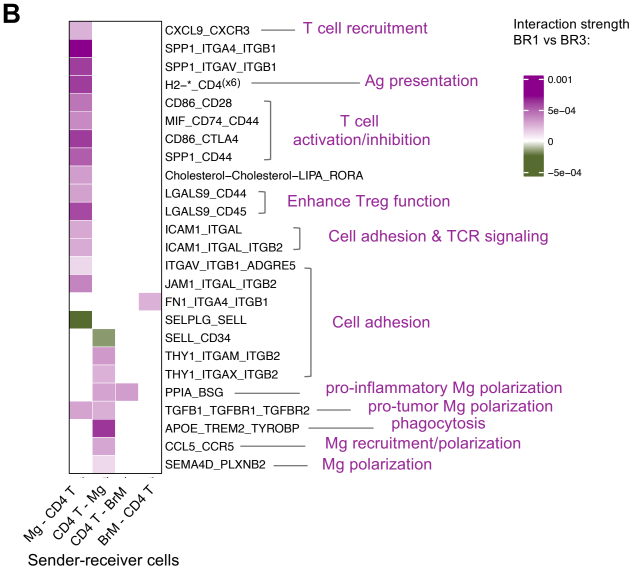

C

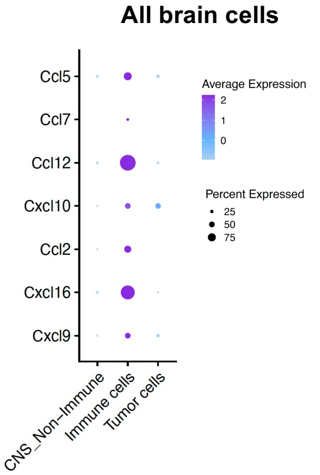

D

Human CD8 T cell clusters	Selected genes	Similar mouse CD8 T cell clusters*
0	*HAVCR2,LAG3,PRF1, ENTPD1*	**CD8.sc5** (“activated/exhausted”)
1	*ENTPD1,TOX,TCF1, CXCL13*	**CD8.sc4** (“exhausted stem cell-like)
2	*PDCD1, GZMA, PRF1, ITGAE*	NA
3	*LAG3,TCF7*	**CD8.sc4** (“exhausted stem cell-like)
4	*HAVCR2,LAG3,TIGIT, CTLA4, IFNG, TOX, ENTPD1*	**CD8.sc5** (“activated/exhausted”)
5	*SELL, CCR7, IL7R, TCF7, GZMK, GZMB, GZMA*	**Mix CD8.sc1 and CD8.sc3** (Naïve and effector memory)
6	*MKI67,CDK1*	**CD8.sc6** (proliferative)
7	*MKI67,CDK1, ENTPD1*	**CD8.sc6** (proliferative)
8	*MKI67, CTLA4, GZMA, GZMK,GZMB, PRF1*	**CD8.sc6** (proliferative)

*Enriched in **BR1** or **BR3**

E

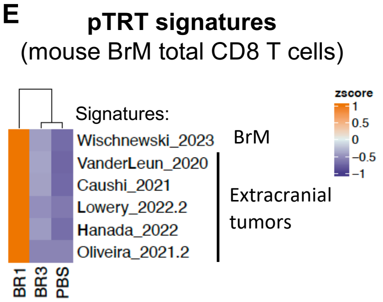

F

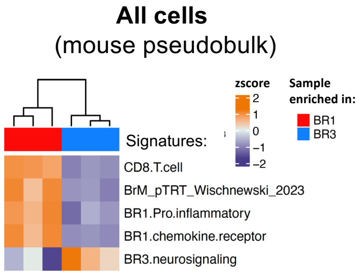

G

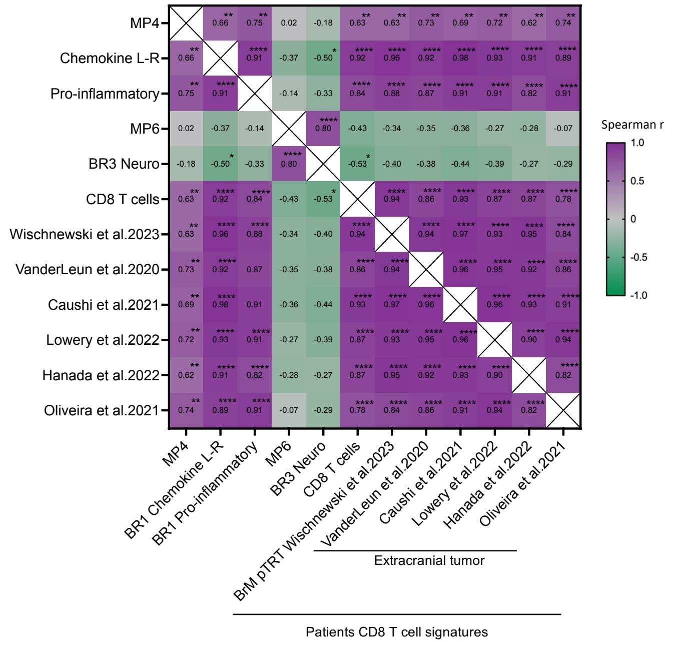

Figure S6: BR1 and BR3 tumor molecular features control the BrTME.

(A-B) Cell-cell interaction analysis from scRNA-seq data of untreated BR1 and BR3 brains. (A) Total number of interactions between indicated cell sender-cell receiver pairs per group. (B) Heatmap showing the top and bottom 10% most differential interaction strength of ligand-receptor (L-R) pairs between BR1 versus BR3 (see methods) for each indicated cell type pair. Interactions stronger in BR1 versus BR3 are depicted in purple, interactions stronger in BR3 depicted in green. Interaction family names in black indicate equal distribution between BR1 and BR3. Numbers in parentheses indicate the number of different L-R pairs. No number indicates that only one L-R pair is present. (C) scRNA-seq analysis of all brain cells from untreated BR1 and BR3-bearing mice. Dot plot showing the expression of selected chemokines in each cell type. (D) Human $CD8^+$ T cell clusters identified in Biermann et al. 2022 dataset with their selected expressed genes and similar mouse $CD8^+$ T cell clusters from BR1 and BR3 models. (E) scRNA-seq analysis of total $CD8^+$ T cells from BR1, BR3, and non-tumor (PBS) bearing mice. Heatmap showing Z-score expression level of various published gene signatures described as "tumor-reactive" CD8 T cells in cancer patients. See supplementary table S6 for details. (F) Pseudobulk RNA-seq analysis of BR1 and BR3-bearing brains. Heatmap showing Z-score expression of $CD8^+$ T cells, BR1, and BR3 signatures among total brain cells across samples. (G) Pseudo-bulk RNA-seq analysis of Sun et al. 2023 pan-cancer BrM patient cohort. Spearman correlation matrix between the different BR1 and BR3 signatures in patient biopsies. See also Table S6.

Table S1: Tumor cell line mutated genes

The number is variant allele frequncy (VAF). Subline-specific mutated genes can be shown by filtering genes with VAF = 0 in other two lines.

In red: unique mutations found in BR1

In blue: unique mutations found in BR3

	parental	BR1	BR3
Abca1	1	1	0.99
Inpp5b	1	0.99	1
Ago4	0.99	1	0.99
Mcf2	0.99	1	1
Prpf38a	0.99	0.99	0.99
Aifm1	0.98	1	1
Plp1	0.94	0.99	1
Rbm19	0.69	0.71	0.65
Irak3	0.68	0.66	0.68
Pilrb1	0.68	0.59	0.66
Baiap2l1	0.66	0.65	0.67
Adamts19	0.65	0.61	0.65
Mcph1	0.65	0.68	0.64
Inpp4b	0.64	0.68	0.64
Lphn1	0.64	0.67	0.67
Trim46	0.64	0.55	0.42
Vdr	0.64	0.58	0.33
Hmgb2	0.63	0.64	0.55
Usp13	0.63	0.54	0.49
Glra3	0.62	0.64	0.52
Ikbkb	0.62	0.69	0.62
Cyp2d34	0.61	0.51	0.32
Scg2	0.6	0.56	0.32
Gimap6	0.57	0.34	0.38
Ikzf3	0.56	0.47	0.53
Dnah6	0.55	0.38	0.36
Gm4636	0.55	0.4	0.53
Vmn1r42	0.55	0.38	0.35
Cul9	0.54	0.49	0.5
Knstrn	0.54	0.47	0.52
Obscn	0.54	0.53	0.48
Sec14l4	0.54	0.46	0.47
Vmn1r8	0.54	0.36	0.34
Alas1	0.53	0.53	0.48
Fam98c	0.53	0.46	0.49
Pepd	0.53	0.5	0.41
Ptchd3	0.53	0.51	0.49
Vmn1r17	0.53	0.32	0.35
1700029F12Rik	0.52	0.47	0.49

SUPPLEMENTARY TABLES

Table S1: Tumor cell line mutated genes.

Table S2: Immune cell annotations.

Table S3: CD8 T cell cluster defining genes.

Table S4: CD4 T cell cluster defining genes.

Table S5: List of the genes associated to each metaprogram.

Table S6: Patient T cell signatures and BR1 and BR3 genes signatures.

Casc5	0.52	0.46	0.46
Gpank1	0.52	0.47	0.5
Il3	0.52	0.5	0.53
Larp1	0.52	0.5	0.5
Lrrn2	0.52	0.47	0.29
Pde11a	0.52	0.47	0.47
Slc22a6	0.52	0.48	0.49
Uggt2	0.52	0.5	0.52
Uso1	0.52	0.54	0.46
Zfp748	0.52	0.29	0.5
Actc1	0.51	0.53	0.44
Atxn7l1	0.51	0.56	0.48
Ebf2	0.51	0.48	0.49
Gas8	0.51	0.54	0.47
Gcfc2	0.51	0.34	0.34
Gm527	0.51	0.63	0.46
Macf1	0.51	0.54	0.47
Ms4a5	0.51	0.47	0.47
Shank1	0.51	0.45	0.48
Snta1	0.51	0.47	0.49
Sp110	0.51	0.5	0.47
Sp140	0.51	0.51	0.52
Abcc4	0.5	0.49	0.55
Acsf3	0.5	0.5	0.52
Actl9	0.5	0.45	0.48
Cd36	0.5	0.47	0.48
Cdh15	0.5	0.48	0.49
Dis3l	0.5	0.49	0.48
Dock9	0.5	0.47	0.49
Erbb4	0.5	0.57	0.32
Fn3k	0.5	0.49	0.5
Gdpd5	0.5	0.49	0.5
Gnaq	0.5	0.49	0.42
Lrdd	0.5	0.51	0.48
Nsmaf	0.5	0.49	0.47
Pde3b	0.5	0.44	0.52
Rab11fip3	0.5	0.47	0.45
Rims1	0.5	0.48	0.48
Tfap2a	0.5	0.45	0.51
Tmeff2	0.5	0.48	0.5
Wdr60	0.5	0.58	0.5
Bai3	0.49	0.47	0.41
Cbx2	0.49	0.52	0.45
Exph5	0.49	0.5	0.5
Fes	0.49	0.51	0.5

Fmn1	0.49	0.49	0.5
Gm12169	0.49	0.47	0.45
Gpr125	0.49	0.47	0.47
Itgad	0.49	0.49	0.45
Mlh3	0.49	0.6	0.55
Ms4a4d	0.49	0.48	0.46
N4bp2	0.49	0.47	0.48
Naip5	0.49	0.45	0.48
Olfr1494	0.49	0.49	0.48
Olfr77	0.49	0.48	0.48
Slc19a1	0.49	0.46	0.51
Tcerg1l	0.49	0.49	0.49
Vmn2r115	0.49	0.46	0.48
7-Mar	0.48	0.46	0.46
2610008E11Rik	0.48	0.54	0.44
A830010M20Rik	0.48	0.48	0.5
Aatk	0.48	0.51	0.52
Atp10a	0.48	0.52	0.51
Bnip3l	0.48	0.43	0.45
C2cd4c	0.48	0.46	0.47
Chek2	0.48	0.47	0.47
Dock8	0.48	0.52	0.49
Hc	0.48	0.45	0.49
Hs3st5	0.48	0.45	0.49
Itgb1	0.48	0.5	0.5
Mctp1	0.48	0.45	0.51
Polr2a	0.48	0.45	0.46
Serpina3k	0.48	0.39	0.47
Vmn2r110	0.48	0.46	0.47
Zfyve27	0.48	0.46	0.46
9030617O03Rik	0.47	0.59	0.44
Acaa2	0.47	0.46	0.46
Anapc1	0.47	0.51	0.47
Col6a2	0.47	0.47	0.51
Col7a1	0.47	0.5	0.5
Dse	0.47	0.48	0.46
Enam	0.47	0.52	0.49
Fat3	0.47	0.49	0.39
Gpr83	0.47	0.53	0.51
Hbp1	0.47	0.39	0.54
Hrasls5	0.47	0.49	0.43
Llgl1	0.47	0.46	0.48
Nlrp4c	0.47	0.51	0.5
Nme9	0.47	0.48	0.47
Olfr1373	0.47	0.5	0.49

Olfr352	0.47	0.47	0.45
Pdcd6ip	0.47	0.46	0.45
Taf5l	0.47	0.51	0.49
Tnks1bp1	0.47	0.46	0.52
Vmn2r97	0.47	0.44	0.47
Zbtb12	0.47	0.46	0.47
Zfp865	0.47	0.46	0.5
Atr	0.46	0.48	0.45
Cdh23	0.46	0.49	0.52
Csf2ra	0.46	0.42	0.44
Fra10ac1	0.46	0.47	0.48
Gm597	0.46	0.46	0.46
Khk	0.46	0.47	0.48
Myh6	0.46	0.43	0.47
Myo9a	0.46	0.47	0.47
Nedd4l	0.46	0.47	0.46
Nom1	0.46	0.46	0.43
Nt5dc1	0.46	0.52	0.41
Olfr1024	0.46	0.5	0.52
Ostm1	0.46	0.5	0.44
Pfas	0.46	0.42	0.49
Phyhipl	0.46	0.52	0.47
Pramel7	0.46	0.5	0.42
Prr14l	0.46	0.45	0.46
Rab26	0.46	0.44	0.48
Abca13	0.45	0.45	0.44
Atp4a	0.45	0.48	0.46
B4galnt4	0.45	0.47	0.46
Ceacam20	0.45	0.45	0.52
Fras1	0.45	0.46	0.52
Gapdhs	0.45	0.55	0.61
Gm12185	0.45	0.43	0.44
Gnaz	0.45	0.47	0.49
Grid1	0.45	0.46	0.44
Gzmd	0.45	0.5	0.47
Map4k4	0.45	0.48	0.49
Mettl9	0.45	0.48	0.43
Olfr1466	0.45	0.5	0.46
Olfr90	0.45	0.5	0.45
Pds5a	0.45	0.47	0.45
Pla2g4d	0.45	0.49	0.49
Ppp1r18	0.45	0.44	0.49
Proser2	0.45	0.5	0.48
Ric8	0.45	0.49	0.44
Rps12	0.45	0.45	0.44

Tmc5	0.45	0.47	0.51
Top2a	0.45	0.5	0.47
Vmn2r124	0.45	0.48	0.44
Zfyve19	0.45	0.5	0.47
Cpeb1	0.44	0.44	0.51
Gm4745	0.44	0.44	0.47
Hoxb2	0.44	0.52	0.56
Mbd5	0.44	0.43	0.46
Mrc1	0.44	0.44	0.45
Ncoa7	0.44	0.45	0.45
Psmd13	0.44	0.45	0.45
Tdrd5	0.44	0.5	0.64
Ttn	0.44	0.48	0.48
5730507C01Rik	0.43	0.35	0.43
9530053A07Rik	0.43	0.5	0.46
Ager	0.43	0.47	0.43
Eef2	0.43	0.44	0.46
Enpp7	0.43	0.44	0.52
Fat2	0.43	0.43	0.45
Gm1110	0.43	0.49	0.5
Khnyn	0.43	0.48	0.56
Med13	0.43	0.48	0.49
Mia2	0.43	0.59	0.45
Prg4	0.43	0.48	0.65
Trp53bp2	0.43	0.46	0.67
Pon3	0.42	0.62	0.49
Astn1	0.41	0.46	0.7
Atp2b4	0.41	0.42	0.63
Kcnj3	0.41	0.5	0.43
Nfxl1	0.41	0.42	0.48
Pkhd1	0.41	0.44	0.53
Sele	0.41	0.49	0.62
Capn3	0.4	0.53	0.47
E330021D16Rik	0.4	0.61	0.62
Mapk7	0.4	0.43	0.45
Aox3	0.39	0.42	0.65
Crhr2	0.39	0.63	0.58
Dnah2	0.39	0.48	0.48
Srebf2	0.39	0.51	0.68
Ubash3b	0.39	0.44	0.46
Tatdn3	0.38	0.43	0.64
Apol7e	0.37	0.43	0.67
Cacna1i	0.37	0.47	0.64
Dennd6b	0.37	0.45	0.62
Lmna	0.37	0.43	0.48

Atp2b1	0.36	0.36	0.31
Cttnbp2	0.36	0.61	0.47
S100a9	0.36	0.45	0.5
Ankhd1	0.35	0.41	0.3
Asic1	0.35	0.46	0.67
Cdh8	0.35	0.29	0.33
Col24a1	0.35	0.44	0.5
Dync1li2	0.35	0.34	0.36
Pdxp	0.35	0.45	0.62
Sf3b1	0.35	0.42	0.6
Sharpin	0.35	0.42	0.68
Tlr2	0.35	0.44	0.48
Chga	0.34	0.41	0.52
Pnpla3	0.34	0.41	0.67
Slc48a1	0.34	0.2	0.31
Sprr2f	0.34	0.41	0.5
Trps1	0.34	0.37	0.61
Vmn2r121	0.34	0.36	0.37
Bai1	0.33	0.26	0.33
Cog4	0.33	0.34	0.32
Mfn1	0.33	0.31	0.49
Nav3	0.33	0.32	0.3
Rsbn1	0.33	0.4	0.52
Slc29a4	0.33	0.26	0.28
Bcar1	0.32	0.31	0.32
Cage1	0.32	0.36	0.37
Ccdc53	0.32	0.34	0.3
Eea1	0.32	0.3	0.29
Gm8298	0.32	0.58	0.43
Scube1	0.32	0.41	0.66
Spag1	0.32	0.29	0.64
Ido2	0.31	0.32	0.36
Nr4a1	0.31	0.42	0.63
Ptpro	0.31	0.3	0.29
Tdpoz3	0.31	0.39	0.45
Trio	0.31	0.37	0.62
Cul2	0.3	0.37	0.32
Gdf1	0.3	0.32	0.47
Irs2	0.3	0.34	0.31
Tbxas1	0.3	0.33	0.31
Adamts20	0.29	0.18	0.29
Kras	0.29	0.28	0.29
Nod1	0.29	0.31	0.28
BC094916	0.28	0.32	0.43
Jhdm1d	0.28	0.27	0.21

Vmn2r18	0.27	0.2	0.31
Cep120	0.26	0.33	0.32
Gm14446	0.26	0.26	0.24
Peg10	0.25	0.29	0.27
Rictor	0.24	0.26	0.32
Vmn1r77	0.24	0.24	0.24
Amfr	0.23	0.17	0.25
Ptpn3	0.23	0.25	0.48
Il17rc	0.21	0.34	0.34
Clec4a2	0.2	0.3	0.32
Cdca3	0.19	0.31	0.3
Gm13154	0.18	0.53	0.42
Vmn2r89	0.17	0.2	0.17
Eri1	0.16	0.24	0.18
Apba3	0.14	0	0
Asmt	0.5	0	0
Atl2	0.19	0	0
Bbs9	0.14	0	0
Bcorl1	0.12	0	0
Clca6	0.34	0	0
Cog2	0.14	0	0
Cst7	0.14	0	0
Cyp2f2	0.13	0	0
Ect2l	0.13	0	0
Irf2bp2	0.13	0	0
Mphosph6	0.16	0	0
Mx2	0.13	0	0
Olfr902	0.16	0	0
Olfr951	0.16	0	0
Peg3	0.15	0	0
Plekho1	0.31	0	0
R3hdm1	0.12	0	0
Slc13a4	0.22	0	0
Smarcc1	0.19	0	0
Snai1	0.16	0	0
Stk32c	0.13	0	0
Tenm2	0.15	0	0
Vps13b	0.13	0	0
Xrn1	0.17	0	0
Zfc3h1	0.12	0	0
2310035C23Rik	0	0.11	0
4632434I11Rik	0	0.23	0
4921506M07Rik	0	0.17	0
4930402H24Rik	0	0.11	0
Abcc2	0	0.14	0

Abcc6	0	0.13	0
Abhd16b	0	0.23	0
Adam19	0	0.21	0
Adamts9	0	0.16	0
Agpat4	0	0.24	0
Ajap1	0	0.25	0
Ak2	0	0.22	0
Akap13	0	0.13	0
Alpk2	0	0.22	0
Alpk3	0	0.1	0
Ankk1	0	0.1	0
Atp13a2	0	0.15	0
Atp2b2	0	0.19	0
Atp6v0d2	0	0.12	0
Atpaf2	0	0.26	0
Bcmo1	0	0.18	0
Cacna1b	0	0.17	0
Ccdc162	0	0.12	0
Ccdc171	0	0.14	0
Ccdc177	0	0.25	0
Ccdc64b	0	0.14	0
Chrm2	0	0.17	0
Ckm	0	0.12	0
Coq9	0	0.12	0
Cpz	0	0.2	0
Csrnp3	0	0.11	0
Cxadr	0	0.2	0
Cyp2b23	0	0.21	0
Diap1	0	0.12	0
Dido1	0	0.19	0
Dlg5	0	0.13	0
Dnah7a	0	0.18	0
Dspp	0	0.12	0
Eif5	0	0.32	0
Elmo2	0	0.13	0
En2	0	0.15	0
Fam179b	0	0.12	0
Fanca	0	0.12	0
Fancg	0	0.12	0
Fbxo40	0	0.21	0
Frem1	0	0.18	0
Fuca1	0	0.11	0
Gab2	0	0.21	0
Galnt3	0	0.24	0
Gm10220	0	0.15	0

Gm6614	0	0.1	0
Greb1	0	0.19	0
Gse1	0	0.13	0
Gstk1	0	0.13	0
Herc4	0	0.24	0
Hiatl1	0	0.1	0
Hnrnpul2	0	0.18	0
Huwe1	0	0.22	0
Il13ra2	0	0.19	0
Il17d	0	0.12	0
Il17rd	0	0.2	0
Il1f10	0	0.13	0
Itgb8	0	0.19	0
Jmy	0	0.22	0
Kcnab2	0	0.23	0
Kcnq5	0	0.19	0
Kcnu1	0	0.15	0
Klf2	0	0.1	0
Klhdc4	0	0.19	0
Kndc1	0	0.24	0
Krt82	0	0.14	0
Lipi	0	0.2	0
Lmf1	0	0.12	0
Ltbp1	0	0.13	0
Mark4	0	0.11	0
Mid1ip1	0	0.28	0
Mllt6	0	0.22	0
Ms4a4c	0	0.13	0
Msh4	0	0.2	0
Muc4	0	0.21	0
Nedd4	0	0.25	0
Nfatc2	0	0.14	0
Nlgn3	0	0.1	0
Npb	0	0.21	0
Nxf2	0	0.49	0
Ogdh	0	0.19	0
Olfr1341	0	0.13	0
Olfr1347	0	0.23	0
Olfr198	0	0.23	0
Olfr304	0	0.24	0
Olfr676	0	0.19	0
Olfr691	0	0.2	0
Olfr911-ps1	0	0.22	0
Paqr3	0	0.09	0
Phf3	0	0.12	0

Phf8	0	0.42	0
Pias2	0	0.23	0
Pikfyve	0	0.19	0
Plec	0	0.12	0
Ppm1g	0	0.18	0
Prkrip1	0	0.15	0
Prr14	0	0.22	0
Prrc2a	0	0.28	0
Prrt4	0	0.12	0
Prss50	0	0.09	0
Rab3gap1	0	0.11	0
Rad21l	0	0.22	0
Rbp3	0	0.13	0
Rdh13	0	0.16	0
Rnf139	0	0.15	0
Rusc2	0	0.21	0
Scube3	0	0.11	0
Serpinb10	0	0.18	0
Sgms2	0	0.14	0
Shroom4	0	0.27	0
Slc2a12	0	0.13	0
Slc35f1	0	0.12	0
Smc2	0	0.18	0
Smurf1	0	0.15	0
Sos1	0	0.14	0
Spopl	0	0.2	0
Stk33	0	0.13	0
Stxbp5l	0	0.11	0
Syt10	0	0.15	0
Sytl2	0	0.11	0
Tcl1b4	0	0.37	0
Tdrd7	0	0.14	0
Trip12	0	0.2	0
Trpm8	0	0.24	0
Unc79	0	0.18	0
Wdhd1	0	0.2	0
Xrra1	0	0.17	0
Zbed6	0	0.22	0
Zfp869	0	0.13	0
1700066M21Rik	0	0	0.32
5330417C22Rik	0	0	0.5
Abcb5	0	0	0.14
Acot12	0	0	0.49
Aim1l	0	0	0.5
Ankrd13d	0	0	0.45

Arhgap29	0	0	0.5
Arhgap44	0	0	0.48
Aspa	0	0	0.14
Atp13a3	0	0	0.99
B020004J07Rik	0	0	0.16
Car3	0	0	0.42
Cast	0	0	0.36
Ccdc55	0	0	0.15
Cd101	0	0	0.45
Cdc25c	0	0	0.24
Cdh26	0	0	0.37
Cep250	0	0	0.46
Chl1	0	0	0.28
Cit	0	0	0.32
Cnot1	0	0	0.31
Coro1c	0	0	0.32
Crb2	0	0	0.45
Csmd3	0	0	0.33
Ctnna2	0	0	0.33
Ctsq	0	0	0.44
Cul5	0	0	0.46
Cyp2t4	0	0	0.5
Dchs1	0	0	0.5
Ddx50	0	0	0.32
Dgkz	0	0	0.46
Dmd	0	0	0.26
Dmtf1	0	0	0.47
Dnah10	0	0	0.32
Doc2a	0	0	0.48
Duoxa1	0	0	0.4
Dync2h1	0	0	0.4
Efcab6	0	0	0.24
Efnb2	0	0	0.34
Epha3	0	0	1
Ephx3	0	0	0.51
Fermt1	0	0	0.5
Fgd6	0	0	0.31
Frmd4a	0	0	0.46
Gcc2	0	0	0.46
Gcg	0	0	0.45
Gm5150	0	0	0.48
Gpr98	0	0	0.5
Gpt2	0	0	0.28
Gtl3	0	0	0.32
Hepacam2	0	0	0.24

Hexa	0	0	0.47
Hibch	0	0	0.47
Hlf	0	0	0.47
Hspa12b	0	0	0.46
Kdm4c	0	0	0.14
Klra6	0	0	0.28
Lao1	0	0	0.43
Lrig3	0	0	0.27
Lrrc52	0	0	0.66
Map2k4	0	0	0.5
Mfn2	0	0	0.45
Mfsd1	0	0	0.51
Midn	0	0	0.45
Mpdz	0	0	0.52
Myh7b	0	0	0.16
Myh8	0	0	0.5
Myo18a	0	0	0.46
Nell1	0	0	0.44
Net1	0	0	0.55
Olfr1116-ps	0	0	0.46
Olfr1263	0	0	0.47
Olfr134	0	0	0.49
Olfr1501	0	0	0.47
Olfr591	0	0	0.47
Olfr776	0	0	0.34
Olfr847	0	0	0.18
Pcdh12	0	0	0.34
Pde3a	0	0	0.22
Pnpt1	0	0	0.5
Ppp1r35	0	0	0.46
Psd4	0	0	0.32
Pten	0	0	0.35
Ptgr1	0	0	0.51
Ptprt	0	0	0.46
Rab10	0	0	0.44
Rad51d	0	0	0.37
Rimbp2	0	0	0.33
Rin2	0	0	0.44
Rps6kb2	0	0	0.48
Scn9a	0	0	0.35
Sh3yl1	0	0	0.53
Skint5	0	0	0.46
Slc12a6	0	0	0.47
Snx24	0	0	0.3
Sppl2c	0	0	0.46

Stx16	0	0	0.45
Syne2	0	0	0.44
Tas2r122	0	0	0.29
Tmem104	0	0	0.48
Tmem132d	0	0	0.3
Tmem177	0	0	0.63
Tmem2	0	0	0.49
Tomm40	0	0	0.44
Trem1	0	0	0.18
Trim71	0	0	0.5
Trip11	0	0	0.44
Tsc1	0	0	0.13
Ubap2	0	0	0.51
Ubtfl1	0	0	0.18
Vim	0	0	0.52
Wdr59	0	0	0.31
Xkr4	0	0	0.19
Zar1	0	0	0.52
Zfp341	0	0	0.53
Zfp831	0	0	0.17
Abca3	0.18	0.23	0
Atp13a5	0.66	0.97	0
Atp6v1a	0.63	0.96	0
Bcor	0.31	0.16	0
Clcn5	0.16	0.22	0
Foxb2	0.16	0.13	0
Gria1	0.18	0.13	0
Hnrnph2	0.32	0.21	0
Igsf5	0.65	0.97	0
Lrrc40	0.22	0.1	0
Pou3f3	0.15	0.12	0
Setx	0.14	0.13	0
Slc4a11	0.16	0.13	0
Sycp2	0.16	0.14	0
Topors	0.17	0.13	0
Ube2dnl2	0.33	0.26	0
Usp9y	0.99	1	0
4930451C15Rik	0.29	0	1
Ccdc14	0.13	0	0.99
Gm8693	0.95	0	0.93
Olfr656	0.2	0	0.45
Olfr820	0.13	0	0.32
Smpd4	0.3	0	1
Dpp10	0	0.22	0.66
Fat1	0	0.14	0.11

Rsf1	0	0.62	0.5

Table S2. Flow cytometry immune cell annotations

Population	Abbreviation	Primary markers used for identification
Infiltrating cells	$CD45^{hi}$	$^{a}Lin^{neg}CD45.2^{hi}CD44^{pos/hi}CD49d^{pos/hi}$
Microglia	Mg	$CD45^{low/+}CD44^{low}CD49d^{low}TMEM119^{+/hi}P2RY12^{low/hi}SiglecH^{low/hi}CX3CR1^{+/hi}CD11b^{+}$
Total T cells	T cells	$^{a}Lin^{neg}CD45.2^{hi}SSC\text{-}A^{low}NK1.1^{neg}CD19^{neg}CD3^{+}$
CD8 T cells	CD8 T	$^{a}Lin^{neg}CD45.2^{hi}SSC\text{-}A^{low}NK1.1^{neg}CD19^{neg}CD3^{+}CD8^{+}CD4^{neg}$
CD4 T cells	CD4 T	$^{a}Lin^{neg}CD45.2^{hi}SSC\text{-}A^{low}NK1.1^{neg}CD19^{neg}CD3^{+}CD8^{neg}CD4^{+}$
$CD8^{+}CD4^{+}$ T cells	DP T	$^{a}Lin^{neg}CD45.2^{hi}SSC\text{-}A^{low}NK1.1^{neg}CD19^{neg}CD3^{+}CD8^{+}CD4^{+}$
$CD8^{-}CD4^{-}$ T cells	DN T	$^{a}Lin^{neg}CD45.2^{hi}SSC\text{-}A^{low}NK1.1^{neg}CD19^{neg}CD3^{+}CD8^{neg}CD4^{neg}$
Natural killer	NK	$^{a}Lin^{neg}CD45.2^{hi}SSC\text{-}A^{low}CD19^{neg}CD3^{neg}NK1.1^{hi}$
Natural killer T cells	NKT	$^{a}Lin^{neg}CD45.2^{hi}SSC\text{-}A^{low}CD19^{neg}CD3^{+}NK1.1^{hi}$
Innate lymphoid cells	ILCs	$^{a}Lin^{neg}CD45.2^{hi}SSC\text{-}A^{low}NK1.1^{neg}CD19^{neg}CD3^{neg}CD11b^{neg}CD11c^{neg}$
B cells	B cells	$^{a}Lin^{neg}CD45.2^{hi}CD3^{neg}NK1.1^{neg}CD19^{+}MHCII^{+}$
Neutrophils	Neu	$^{b}Lin^{neg}CD45.2^{hi}SSC\text{-}A^{int}CD11b^{+}Ly6C^{int}Ly6G^{+}$
Mononuclear phagocytes	Mononuclear phagocytes	$^{b}Lin^{neg}CD45.2^{hi}CD44^{+/hi}CD49d^{hi}Ly6G^{neg}CD11b^{+}$<br>$^{b}Lin^{neg}CD45.2^{hi}CD44^{+/hi}CD49d^{hi}Ly6G^{neg}CD11c^{+}$
Classical Monocytes	$Ly6C^{hi}$ Monocytes	$^{b}Lin^{neg}Ly6G^{neg}CX3CR1^{+}CD44^{hi}F4/80^{neg\text{-}low}CD11c^{neg}MHCII^{neg}Ly6C^{hi}$
Non-classical Monocytes	$Ly6C^{low}$ Monocytes	$^{b}Lin^{neg}Ly6G^{neg}CX3CR1^{+}CD44^{hi}F4/80^{low/int}CD11c^{low}MHCII^{neg}Ly6C^{low}$
Monocyte-derived	Monoderived	$^{b}Lin^{neg}CD45.2^{hi}Ly6G^{neg}CD11b^{int/hi}CD11c^{neg\text{-}low\text{-}int}SiglecH^{neg}CD24^{neg}MHCII^{+/hi}MertK^{pos/hi}F4/80^{pos/hi}$
Dendritic cells	DC	$^{a}Lin^{neg}Ly6G^{neg}CD11c^{+}MHCII^{+}F4/80^{neg}CD24^{+}$
Dendritic cells 1	DC1	$^{a}Lin^{neg}Ly6G^{neg}CD11c^{hi}MHCII^{+}F4/80^{neg}CD11b^{neg}CD24^{hi}CD103^{hi}$
Dendritic cells 2	DC2	$^{a}Lin^{neg}Ly6G^{neg}CD11c^{+}MHCII^{hi}F4/80^{neg}CD11b^{+}CD24^{neg}CD103^{neg}$
Dendritic cells 3	DC.pop3	$^{a}Lin^{neg}Ly6G^{neg}CD11c^{+}MHCII^{+}F4/80^{neg}CD11b^{neg}CD24^{hi}CD103^{neg}$
Plasmacytoid DC	pDC	$^{a}Lin^{neg}Ly6G^{neg}CD11c^{int}MHCII^{lo}Ly6C^{+}SiglecH^{+}$

$^{a}Lin^{neg}$: $TMEM119^{neg}P2RY12^{neg}$
$^{b}Lin^{neg}$: $TMEM119^{neg}P2RY12^{neg}NK1.1^{neg}CD3^{neg}CD19^{neg}$

Table S3: CD8 T cell cluster defining genes

p_val	avg_log 2FC	pct.1	pct.2	p_val_adj	cluster	cluster name	gene
1.95E-68	1.931	0.983	0.550	5.11E-64	0	sc.5	S100a6
2.54E-66	1.235	1.000	0.817	6.67E-62	0	sc.5	Cd3g
2.61E-65	1.362	1.000	0.788	6.85E-61	0	sc.5	Nkg7
9.43E-62	1.314	1.000	0.790	2.47E-57	0	sc.5	AW112010
9.58E-62	1.411	1.000	0.581	2.51E-57	0	sc.5	Cxcr6
1.55E-60	2.079	0.991	0.658	4.05E-56	0	sc.5	Ccl5
2.26E-59	1.733	0.945	0.396	5.92E-55	0	sc.5	S100a4
3.96E-59	1.364	0.766	0.162	1.04E-54	0	sc.5	Gm36723
6.75E-52	1.044	0.830	0.254	1.77E-47	0	sc.5	Fgl2
2.33E-49	1.852	0.770	0.223	6.10E-45	0	sc.5	Gzmb
2.16E-48	0.979	0.996	0.750	5.67E-44	0	sc.5	Cd8a
1.43E-47	1.160	0.689	0.175	3.75E-43	0	sc.5	Tcrg-C2
7.43E-47	1.383	0.923	0.406	1.95E-42	0	sc.5	Ccl4
6.27E-46	1.055	0.953	0.642	1.64E-41	0	sc.5	S100a11
2.05E-43	1.104	0.983	0.565	5.36E-39	0	sc.5	Id2
4.23E-43	1.288	0.932	0.633	1.11E-38	0	sc.5	Klrd1
7.42E-42	0.948	0.987	0.571	1.95E-37	0	sc.5	Rbpj
1.46E-41	0.941	0.932	0.429	3.84E-37	0	sc.5	Pdcd1
1.95E-41	1.196	0.983	0.681	5.12E-37	0	sc.5	Lgals1
1.06E-39	1.087	0.677	0.202	2.78E-35	0	sc.5	Trgv2
1.23E-37	0.933	0.783	0.292	3.24E-33	0	sc.5	Lag3
2.82E-37	0.949	0.685	0.192	7.39E-33	0	sc.5	Havcr2
2.16E-35	0.935	0.647	0.200	5.66E-31	0	sc.5	Cd200r1
3.43E-35	1.140	0.651	0.192	9.00E-31	0	sc.5	Ccl3
1.97E-33	0.941	0.877	0.467	5.16E-29	0	sc.5	Cdk6
1.02E-30	1.011	0.728	0.294	2.68E-26	0	sc.5	Prf1
7.87E-28	1.052	0.553	0.181	2.06E-23	0	sc.5	Klre1
1.80E-27	1.007	0.685	0.277	4.72E-23	0	sc.5	Ifng
1.69E-22	0.955	0.749	0.375	4.44E-18	0	sc.5	Ctla4
1.20E-16	0.956	0.677	0.419	3.14E-12	0	sc.5	Plac8
7.42E-22	1.600	0.639	0.290	1.95E-17	1	sc.3	Ly6c2
1.22E-20	0.629	0.614	0.241	3.20E-16	1	sc.3	Cxcr3
1.91E-16	0.638	0.975	0.791	5.02E-12	1	sc.3	Hcst
1.07E-15	0.617	0.772	0.425	2.82E-11	1	sc.3	Gramd3
1.96E-15	0.561	0.810	0.564	5.15E-11	1	sc.3	Smap1
1.33E-14	0.537	0.709	0.392	3.48E-10	1	sc.3	Hopx
2.46E-14	0.454	1.000	0.993	6.46E-10	1	sc.3	Rpl13a
3.79E-14	0.457	0.994	0.700	9.95E-10	1	sc.3	Ccl5

6.85E-14	0.732	0.854	0.621	1.80E-09	1	sc.3	Zfp36l2
1.04E-13	0.795	0.918	0.794	2.72E-09	1	sc.3	Itgb1
2.79E-13	0.490	1.000	0.992	7.31E-09	1	sc.3	Rps27
3.63E-13	0.593	0.962	0.844	9.52E-09	1	sc.3	Gimap4
1.11E-11	0.497	0.956	0.901	2.92E-07	1	sc.3	Ly6e
1.56E-11	0.526	1.000	0.987	4.09E-07	1	sc.3	Tmsb10
6.09E-11	0.449	0.981	0.814	1.60E-06	1	sc.3	Ptpn18
6.78E-11	0.581	0.892	0.786	1.78E-06	1	sc.3	Ifngr1
1.30E-10	0.561	0.949	0.841	3.41E-06	1	sc.3	Epsti1
2.28E-10	0.531	0.842	0.595	5.98E-06	1	sc.3	Gm2682
4.01E-10	0.490	0.880	0.626	1.05E-05	1	sc.3	Gimap7
6.25E-10	0.528	0.570	0.298	1.64E-05	1	sc.3	Gzmk
8.08E-10	0.440	0.506	0.260	2.12E-05	1	sc.3	Rtp4
1.18E-09	0.563	0.627	0.410	3.09E-05	1	sc.3	Slfn1
1.70E-09	0.451	0.709	0.513	4.46E-05	1	sc.3	Sorl1
3.38E-09	0.437	0.728	0.516	8.85E-05	1	sc.3	Sp110
4.12E-09	0.436	0.905	0.797	0.000	1	sc.3	Gimap3
1.01E-08	0.453	0.994	0.945	0.000	1	sc.3	Ms4a4b
3.58E-08	0.709	0.772	0.591	0.001	1	sc.3	Ly6a
3.98E-08	0.461	0.753	0.538	0.001	1	sc.3	Txnip
6.03E-08	0.495	0.684	0.471	0.002	1	sc.3	Slco3a1
1.31E-55	1.145	0.866	0.186	3.45E-51	2	sc.2	Cd81
1.62E-54	1.324	0.843	0.170	4.25E-50	2	sc.2	Sparc
2.40E-49	1.119	0.827	0.180	6.29E-45	2	sc.2	Sparcl1
1.28E-45	1.068	0.764	0.161	3.35E-41	2	sc.2	Ctsl
1.06E-41	1.374	0.858	0.304	2.78E-37	2	sc.2	Mt1
6.87E-40	1.173	0.835	0.272	1.80E-35	2	sc.2	Ckb
1.37E-38	1.117	0.906	0.346	3.60E-34	2	sc.2	Qk
2.72E-38	1.808	0.984	0.973	7.13E-34	2	sc.2	mt-Nd1
5.42E-37	1.498	1.000	1.000	1.42E-32	2	sc.2	mt-Co1
7.78E-37	1.540	1.000	0.984	2.04E-32	2	sc.2	mt-Nd2
1.05E-36	1.736	1.000	0.994	2.76E-32	2	sc.2	mt-Nd4
1.10E-36	1.084	0.819	0.245	2.89E-32	2	sc.2	Cd63
1.61E-36	1.578	1.000	1.000	4.21E-32	2	sc.2	mt-Atp6
2.87E-35	1.566	1.000	1.000	7.52E-31	2	sc.2	mt-Cytb
5.45E-35	1.099	0.874	0.336	1.43E-30	2	sc.2	C1qb
6.74E-35	1.676	1.000	1.000	1.77E-30	2	sc.2	mt-Co2
4.97E-33	1.568	1.000	1.000	1.30E-28	2	sc.2	mt-Co3
9.81E-33	1.282	0.984	0.682	2.57E-28	2	sc.2	Apoe
1.76E-32	1.027	0.866	0.374	4.61E-28	2	sc.2	C1qa
7.42E-32	1.477	0.992	0.846	1.95E-27	2	sc.2	Ttr
6.28E-31	1.314	0.992	0.811	1.65E-26	2	sc.2	Macf1
2.72E-26	1.213	0.992	0.954	7.14E-22	2	sc.2	AY036118

4.96E-21	1.076	0.984	0.893	1.30E-16	2	sc.2	Cst3
6.15E-21	1.237	0.969	0.667	1.61E-16	2	sc.2	Zbtb20
1.67E-20	1.218	0.992	0.978	4.38E-16	2	sc.2	Camk1d
4.60E-17	1.064	0.992	0.900	1.21E-12	2	sc.2	Elmo1
6.89E-17	1.055	0.969	0.775	1.81E-12	2	sc.2	Gphn
4.70E-15	1.165	0.843	0.621	1.23E-10	2	sc.2	Nr3c1
9.94E-15	1.090	0.945	0.868	2.61E-10	2	sc.2	mt-Nd5
1.21E-10	1.043	0.976	0.882	3.17E-06	2	sc.2	Xist
#######	2.066	0.886	0.043	1.15E-109	3	sc.1	Sell
1.23E-90	1.827	0.927	0.111	3.23E-86	3	sc.1	Cmah
2.59E-89	1.253	0.740	0.035	6.78E-85	3	sc.1	Ccr7
2.08E-84	2.628	0.984	0.209	5.45E-80	3	sc.1	Lef1
1.12E-83	2.250	0.959	0.139	2.93E-79	3	sc.1	Klf2
1.74E-77	1.778	0.967	0.188	4.55E-73	3	sc.1	Tcf7
1.28E-73	2.291	0.943	0.220	3.36E-69	3	sc.1	Satb1
1.62E-65	1.349	0.862	0.150	4.24E-61	3	sc.1	S1pr1
1.66E-65	1.829	0.935	0.241	4.35E-61	3	sc.1	Txk
1.12E-64	1.471	0.837	0.139	2.95E-60	3	sc.1	Klf3
3.84E-60	1.448	0.959	0.220	1.01E-55	3	sc.1	Aff3
2.27E-58	1.832	0.821	0.185	5.95E-54	3	sc.1	Fam241a
1.24E-55	1.273	0.854	0.166	3.26E-51	3	sc.1	Slamf6
1.78E-53	1.676	0.951	0.301	4.68E-49	3	sc.1	Bach2
1.67E-49	1.126	1.000	1.000	4.37E-45	3	sc.1	Rps29
5.83E-49	1.556	0.984	0.710	1.53E-44	3	sc.1	Foxp1
3.10E-47	1.844	0.927	0.391	8.12E-43	3	sc.1	Il7r
2.97E-46	1.667	0.967	0.483	7.78E-42	3	sc.1	Ripor2
9.54E-46	1.450	0.870	0.231	2.50E-41	3	sc.1	Fgf13
3.22E-45	1.426	0.943	0.337	8.45E-41	3	sc.1	Emb
7.15E-44	1.712	0.862	0.323	1.88E-39	3	sc.1	Sidt1
8.78E-44	1.197	0.919	0.421	2.30E-39	3	sc.1	Dgka
5.35E-43	1.623	0.992	0.579	1.40E-38	3	sc.1	Gm2682
1.00E-41	1.081	0.992	0.994	2.62E-37	3	sc.1	Rps19
3.64E-40	1.329	0.764	0.236	9.55E-36	3	sc.1	Rras2
1.96E-38	1.346	0.902	0.380	5.14E-34	3	sc.1	Scml4
1.56E-34	1.231	0.959	0.642	4.09E-30	3	sc.1	Crlf3
1.80E-33	1.154	1.000	0.967	4.72E-29	3	sc.1	Arhgap15
2.79E-31	1.080	0.959	0.616	7.32E-27	3	sc.1	Ssh2
1.23E-26	1.110	0.902	0.419	3.23E-22	3	sc.1	Gramd3
4.72E-47	3.287	0.838	0.166	1.24E-42	4	sc.4	Xcl1
1.73E-34	1.333	0.865	0.236	4.53E-30	4	sc.4	Cd160
2.83E-17	1.037	0.878	0.476	7.41E-13	4	sc.4	Ybx3
9.62E-14	0.921	0.716	0.338	2.52E-09	4	sc.4	Bcl2a1d
3.24E-12	1.158	0.716	0.407	8.50E-08	4	sc.4	Il18r1

1.53E-11	0.918	0.649	0.292	4.02E-07	4	sc.4	Tnfrsf9
3.20E-11	0.729	0.635	0.276	8.40E-07	4	sc.4	Il18rap
3.24E-11	0.584	0.689	0.338	8.49E-07	4	sc.4	Fam162a
5.44E-11	0.800	0.649	0.294	1.43E-06	4	sc.4	Alcam
6.70E-11	0.587	0.878	0.540	1.76E-06	4	sc.4	Elk3
1.05E-10	0.846	0.622	0.302	2.74E-06	4	sc.4	Phactr2
2.15E-10	0.645	0.986	0.809	5.63E-06	4	sc.4	Cd8a
2.55E-10	0.964	0.959	0.758	6.69E-06	4	sc.4	Ifi27l2a
2.85E-10	0.746	0.824	0.496	7.48E-06	4	sc.4	Bcl2a1b
1.11E-09	1.041	0.797	0.499	2.90E-05	4	sc.4	St6galnac3
1.19E-09	0.759	0.838	0.562	3.12E-05	4	sc.4	Nfat5
1.22E-09	0.627	0.757	0.417	3.21E-05	4	sc.4	Cd44
1.53E-09	0.667	0.838	0.512	4.01E-05	4	sc.4	Adamts6
2.57E-09	0.611	0.757	0.411	6.74E-05	4	sc.4	Lag3
4.27E-09	0.679	0.986	0.863	1.119E-04	4	sc.4	Rabgap1l
4.79E-09	0.704	0.932	0.675	1.255E-04	4	sc.4	Rbpj
7.63E-09	0.589	1.000	0.993	2.001E-04	4	sc.4	Hsp90ab1
8.38E-09	0.795	0.608	0.285	2.198E-04	4	sc.4	Rgs16
9.43E-09	0.789	0.649	0.385	2.474E-04	4	sc.4	Nrp1
2.13E-08	0.595	0.959	0.812	5.592E-04	4	sc.4	Eif5a
3.04E-08	0.581	0.824	0.592	7.972E-04	4	sc.4	Trps1
3.80E-08	0.684	0.986	0.802	9.962E-04	4	sc.4	Itgb1
1.80E-07	0.603	0.568	0.297	4.729E-03	4	sc.4	Gpm6b
2.91E-07	0.600	0.824	0.537	7.634E-03	4	sc.4	Mdfic
7.11E-96	3.221	1.000	0.043	1.87E-91	5	sc.6	Hist1h3c
1.57E-94	3.143	0.974	0.040	4.11E-90	5	sc.6	Hist1h2ab
5.21E-78	2.336	1.000	0.063	1.37E-73	5	sc.6	Birc5
7.01E-76	2.096	1.000	0.066	1.84E-71	5	sc.6	Cdca8
1.60E-72	2.753	1.000	0.074	4.20E-68	5	sc.6	Pclaf
3.11E-72	2.367	0.974	0.067	8.16E-68	5	sc.6	Nusap1
7.96E-69	2.104	0.921	0.064	2.09E-64	5	sc.6	Hist1h3f
7.78E-65	2.006	0.974	0.081	2.04E-60	5	sc.6	Kif11
4.16E-56	2.658	0.974	0.117	1.09E-51	5	sc.6	Hist1h3e
2.28E-55	2.500	0.789	0.054	5.99E-51	5	sc.6	Cenpf
3.95E-52	2.195	0.974	0.117	1.04E-47	5	sc.6	Prc1
1.04E-49	2.671	0.816	0.074	2.73E-45	5	sc.6	Ube2c
5.00E-49	2.215	0.974	0.142	1.31E-44	5	sc.6	Hist1h4h
5.70E-49	2.465	0.974	0.148	1.50E-44	5	sc.6	Hist2h2ac
7.13E-48	4.304	1.000	0.169	1.87E-43	5	sc.6	Hist1h1b
5.15E-46	2.696	1.000	0.165	1.35E-41	5	sc.6	Mki67
1.73E-44	4.493	1.000	0.191	4.55E-40	5	sc.6	Hist1h2ae
2.73E-40	1.990	1.000	0.209	7.16E-36	5	sc.6	Smc2
1.39E-38	2.888	1.000	0.229	3.64E-34	5	sc.6	Stmn1

7.12E-37	2.137	0.947	0.185	1.87E-32	5	sc.6	Tubb4b
7.40E-37	3.210	1.000	0.264	1.94E-32	5	sc.6	Top2a
8.30E-37	2.115	0.921	0.183	2.18E-32	5	sc.6	H2afx
6.29E-36	4.842	1.000	0.290	1.65E-31	5	sc.6	Hist1h2ap
1.43E-31	2.358	1.000	0.365	3.76E-27	5	sc.6	Tuba1b
7.01E-29	3.416	1.000	0.497	1.84E-24	5	sc.6	Hist1h4d
6.92E-27	2.571	1.000	0.540	1.82E-22	5	sc.6	Hist1h1e
6.78E-26	2.500	1.000	0.656	1.78E-21	5	sc.6	Tubb5
3.00E-25	3.335	1.000	0.706	7.87E-21	5	sc.6	Hmgb2
5.01E-25	1.976	1.000	0.494	1.32E-20	5	sc.6	Ube2s
2.55E-24	2.124	1.000	0.859	6.69E-20	5	sc.6	H2afz

Table S4: CD4 T cell cluster defining genes

p_val	avg_log2FC	pct.1	pct.2	p_val_adj	cluster	cluster name	gene
2.70E-74	2.4658	0.972	0.382	7.09E-70	0	CD4.sc1	Lef1
4.65E-70	2.2771	0.983	0.299	1.22E-65	0	CD4.sc1	Klf2
1.81E-66	2.4356	0.932	0.312	4.75E-62	0	CD4.sc1	Satb1
2.37E-62	1.4292	0.864	0.151	6.21E-58	0	CD4.sc1	Sell
2.69E-62	2.1106	1	0.497	7.04E-58	0	CD4.sc1	Gm2682
4.68E-62	2.0159	0.994	0.59	1.23E-57	0	CD4.sc1	Foxp1
9.41E-62	1.8122	0.909	0.246	2.47E-57	0	CD4.sc1	Txk
1.29E-58	1.5158	0.938	0.281	3.39E-54	0	CD4.sc1	S1pr1
2.65E-58	1.92	0.926	0.354	6.95E-54	0	CD4.sc1	Bach2
6.09E-55	1.0629	1	1	1.60E-50	0	CD4.sc1	Rps29
2.41E-52	0.9964	0.67	0.078	6.33E-48	0	CD4.sc1	Pik3ip1
2.75E-51	1.5112	0.926	0.374	7.20E-47	0	CD4.sc1	Tcf7
5.81E-48	1.1393	0.71	0.133	1.52E-43	0	CD4.sc1	Actn1
9.80E-48	1.4496	0.972	0.698	2.57E-43	0	CD4.sc1	Crlf3
2.28E-47	1.2499	0.812	0.214	5.99E-43	0	CD4.sc1	Klf3
5.35E-46	1.1001	0.591	0.058	1.40E-41	0	CD4.sc1	Rflnb
3.98E-45	1.4041	0.972	0.636	1.04E-40	0	CD4.sc1	Ripor2
1.43E-44	1.3212	1	0.965	3.74E-40	0	CD4.sc1	Arhgap15
2.40E-44	1.6137	0.506	0.03	6.29E-40	0	CD4.sc1	Igfbp4
8.62E-44	1.143	0.545	0.048	2.26E-39	0	CD4.sc1	St8sia6
6.04E-43	1.0328	0.71	0.136	1.58E-38	0	CD4.sc1	Ccr7
1.23E-42	1.1636	0.932	0.497	3.23E-38	0	CD4.sc1	Dgka
1.35E-40	1.2443	0.858	0.364	3.55E-36	0	CD4.sc1	Scml4
9.21E-40	1.3161	0.778	0.249	2.42E-35	0	CD4.sc1	Cmah
6.09E-35	1.2343	0.835	0.397	1.60E-30	0	CD4.sc1	Tmem71
1.56E-34	1.1863	0.955	0.678	4.09E-30	0	CD4.sc1	Prkcq
8.82E-34	1.0426	0.977	0.907	2.31E-29	0	CD4.sc1	Rapgef6
1.68E-33	1.1313	0.761	0.266	4.41E-29	0	CD4.sc1	St6gal1
2.80E-27	1.0327	0.943	0.751	7.34E-23	0	CD4.sc1	Ikzf1
1.48E-26	1.0186	0.688	0.271	3.88E-22	0	CD4.sc1	Ugcg
1.45E-69	2.6831	0.981	0.296	3.79E-65	1	CD4.sc5	Ikzf2
6.35E-65	1.5118	0.786	0.077	1.66E-60	1	CD4.sc5	Foxp3
6.43E-53	1.8706	0.78	0.14	1.69E-48	1	CD4.sc5	Tnfrsf9
6.99E-47	1.6338	0.761	0.154	1.83E-42	1	CD4.sc5	Il2ra
2.77E-46	1.833	0.95	0.361	7.26E-42	1	CD4.sc5	Tnfrsf4
1.39E-45	1.5504	0.969	0.513	3.65E-41	1	CD4.sc5	Tnfrsf18
3.04E-44	1.208	0.629	0.087	7.97E-40	1	CD4.sc5	Neb
1.90E-38	1.6722	0.962	0.448	5.00E-34	1	CD4.sc5	Ctla4
1.32E-35	1.4683	0.78	0.318	3.46E-31	1	CD4.sc5	Arl5a
4.44E-35	1.3279	0.824	0.34	1.16E-30	1	CD4.sc5	Izumo1r
2.70E-32	1.0323	0.547	0.089	7.08E-28	1	CD4.sc5	Itgb8

3.08E-32	1.1847	0.874	0.414	8.09E-28	1	CD4.sc5	Capg
7.62E-31	1.0991	0.792	0.318	2.00E-26	1	CD4.sc5	Tnfrsf1b
4.00E-30	1.0045	0.906	0.407	1.05E-25	1	CD4.sc5	Il2rb
7.21E-29	1.3823	0.824	0.402	1.89E-24	1	CD4.sc5	Itgav
1.83E-28	1.1439	0.855	0.508	4.79E-24	1	CD4.sc5	Gimap7
1.02E-27	1.2783	0.736	0.323	2.67E-23	1	CD4.sc5	Ighm
4.78E-27	1.3767	0.975	0.848	1.25E-22	1	CD4.sc5	Ifi27l2a
1.40E-26	0.9938	0.906	0.571	3.66E-22	1	CD4.sc5	Pim1
2.49E-23	1.0318	0.881	0.525	6.53E-19	1	CD4.sc5	Samsn1
3.30E-21	1.1678	0.717	0.337	8.66E-17	1	CD4.sc5	Hopx
2.02E-20	0.9867	0.811	0.431	5.29E-16	1	CD4.sc5	Maf
6.45E-20	1.0151	0.969	0.798	1.69E-15	1	CD4.sc5	Rabgap1l
8.10E-19	1.0454	0.912	0.713	2.12E-14	1	CD4.sc5	Sdf4
1.59E-18	1.1913	0.818	0.453	4.17E-14	1	CD4.sc5	S100a6
1.14E-17	0.9536	0.566	0.214	3.00E-13	1	CD4.sc5	Iigp1
1.60E-17	0.9873	0.748	0.373	4.21E-13	1	CD4.sc5	Odc1
1.91E-15	0.9527	0.893	0.672	5.01E-11	1	CD4.sc5	Ly6a
2.28E-15	0.9349	0.881	0.646	5.98E-11	1	CD4.sc5	Icos
2.97E-12	0.9491	0.836	0.677	7.80E-08	1	CD4.sc5	Vim
1.04E-13	0.69	0.672	0.345	2.73E-09	2	CD4.sc2	Arhgap26
6.88E-09	0.5019	0.513	0.262	0.00018	2	CD4.sc2	Cd63
1.06E-07	0.6665	0.807	0.582	0.00277	2	CD4.sc2	Itgb1
2.40E-07	0.7834	0.756	0.582	0.006293	2	CD4.sc2	Emb
4.60E-07	0.635	0.697	0.519	0.012059	2	CD4.sc2	Pde7a
6.15E-07	0.4858	0.958	0.879	0.016137	2	CD4.sc2	Celf2
2.18E-06	0.6497	0.723	0.569	0.05709	2	CD4.sc2	Btg2
3.53E-06	0.7988	0.605	0.378	0.092528	2	CD4.sc2	C1qb
4.44E-42	2.1955	0.965	0.203	1.16E-37	3	CD4.sc3	Cxcr6
6.45E-30	2.1697	0.965	0.311	1.69E-25	3	CD4.sc3	Nkg7
3.56E-25	3.0164	0.895	0.331	9.35E-21	3	CD4.sc3	Ccl5
8.47E-24	1.6638	0.544	0.085	2.22E-19	3	CD4.sc3	Ifng
1.25E-21	1.1158	0.579	0.104	3.29E-17	3	CD4.sc3	Tmem163
1.65E-21	1.5962	0.912	0.427	4.33E-17	3	CD4.sc3	Id2
9.99E-21	1.3405	0.86	0.294	2.62E-16	3	CD4.sc3	Pdcd1
2.02E-18	1.081	1	0.954	5.31E-14	3	CD4.sc3	Cd3g
8.21E-18	1.2564	0.93	0.538	2.15E-13	3	CD4.sc3	Rbpj
5.91E-17	1.6558	0.877	0.414	1.55E-12	3	CD4.sc3	S100a4
6.78E-17	1.3834	1	0.814	1.78E-12	3	CD4.sc3	AW112010
2.36E-15	1.0615	0.825	0.346	6.20E-11	3	CD4.sc3	Hip1
2.96E-15	1.3437	0.772	0.304	7.78E-11	3	CD4.sc3	Dusp2
9.25E-15	0.8408	0.684	0.244	2.43E-10	3	CD4.sc3	Tnfsf10
1.25E-14	1.9371	0.509	0.13	3.27E-10	3	CD4.sc3	Ccl4
4.34E-14	0.9849	0.561	0.159	1.14E-09	3	CD4.sc3	Rgs16
4.95E-14	1.3471	0.86	0.453	1.30E-09	3	CD4.sc3	Rgs1
7.77E-14	1.032	0.947	0.578	2.04E-09	3	CD4.sc3	AU020206

6.53E-13	1.2485	0.947	0.511	1.71E-08	3	CD4.sc3	S100a6
1.62E-12	0.9067	1	0.662	4.26E-08	3	CD4.sc3	S100a11
5.45E-12	0.8826	1	0.973	1.43E-07	3	CD4.sc3	Cd52
6.76E-12	0.9882	0.684	0.273	1.77E-07	3	CD4.sc3	Bhlhe40
1.43E-11	0.972	0.982	0.677	3.75E-07	3	CD4.sc3	Lgals1
7.31E-11	0.7638	0.877	0.507	1.92E-06	3	CD4.sc3	Gng2
6.32E-10	0.7972	0.544	0.193	1.66E-05	3	CD4.sc3	Lgals3
7.11E-10	0.9605	0.807	0.472	1.86E-05	3	CD4.sc3	Cd226
7.43E-10	0.7606	1	0.901	1.95E-05	3	CD4.sc3	Arl6ip1
7.47E-08	1.1071	0.649	0.338	0.001958	3	CD4.sc3	Ctla2a
1.15E-07	0.7851	0.965	0.865	0.003019	3	CD4.sc3	Sub1
1.62E-07	0.7853	0.649	0.354	0.004261	3	CD4.sc3	Ctsw
6.60E-39	1.395	0.775	0.079	1.73E-34	4	CD4.sc4	Gm49180
1.69E-29	2.5873	0.95	0.257	4.44E-25	4	CD4.sc4	Tnfsf8
1.13E-25	1.6995	0.8	0.157	2.96E-21	4	CD4.sc4	Tnfsf11
7.07E-20	1.4357	0.875	0.272	1.85E-15	4	CD4.sc4	Bhlhe40
7.14E-20	1.9977	0.925	0.378	1.87E-15	4	CD4.sc4	Eea1
1.70E-17	1.7789	0.975	0.515	4.47E-13	4	CD4.sc4	Trps1
2.27E-17	1.5468	0.825	0.303	5.96E-13	4	CD4.sc4	Tbc1d4
5.60E-17	1.6436	0.95	0.446	1.47E-12	4	CD4.sc4	Gm15283
6.28E-17	1.3698	0.75	0.2	1.65E-12	4	CD4.sc4	Mir155hg
1.03E-16	1.4134	0.825	0.281	2.71E-12	4	CD4.sc4	Phactr2
9.60E-16	1.5897	0.95	0.566	2.52E-11	4	CD4.sc4	Arap2
4.14E-15	1.5608	0.95	0.691	1.09E-10	4	CD4.sc4	Hif1a
1.06E-13	1.1862	0.8	0.264	2.77E-09	4	CD4.sc4	Kcnq5
1.57E-13	1.1136	0.675	0.202	4.12E-09	4	CD4.sc4	Cd200
4.69E-13	1.1912	0.925	0.537	1.23E-08	4	CD4.sc4	Itpr1
5.07E-13	1.3477	0.9	0.446	1.33E-08	4	CD4.sc4	Odc1
7.10E-13	1.1081	0.925	0.545	1.86E-08	4	CD4.sc4	Jak2
7.71E-13	1.2137	0.95	0.624	2.02E-08	4	CD4.sc4	Ostf1
2.00E-12	1.0762	0.9	0.478	5.24E-08	4	CD4.sc4	Etv6
2.17E-12	1.2973	0.975	0.547	5.69E-08	4	CD4.sc4	Rbpj
2.60E-12	1.1922	0.875	0.459	6.82E-08	4	CD4.sc4	Cd82
5.42E-12	1.2641	0.95	0.755	1.42E-07	4	CD4.sc4	Sdf4
1.69E-11	1.0617	0.95	0.605	4.42E-07	4	CD4.sc4	Itgb1
1.73E-11	1.288	0.625	0.221	4.54E-07	4	CD4.sc4	Gpm6b
2.00E-11	1.0825	0.925	0.479	5.24E-07	4	CD4.sc4	Nfat5
1.60E-10	1.195	0.9	0.513	4.19E-06	4	CD4.sc4	Slamf6
1.80E-10	1.3157	0.975	0.717	4.71E-06	4	CD4.sc4	Stat4
2.92E-10	1.1008	0.775	0.375	7.67E-06	4	CD4.sc4	Kdm2b
7.90E-09	1.1523	0.95	0.764	0.000207	4	CD4.sc4	Prkca
3.18E-07	1.1287	0.675	0.363	0.008349	4	CD4.sc4	Nrp1
1.43E-74	2.8862	1	0.031	3.76E-70	5	CD4.sc6	Hist1h2ab
6.56E-68	2.311	1	0.038	1.72E-63	5	CD4.sc6	Nusap1
5.05E-63	1.9574	0.957	0.038	1.33E-58	5	CD4.sc6	Tpx2

5.27E-61	2.8896	1	0.049	1.38E-56	5	CD4.sc6	Hist1h3c
3.61E-60	2.0618	1	0.049	9.48E-56	5	CD4.sc6	Kif11
1.30E-53	2.0623	0.87	0.038	3.41E-49	5	CD4.sc6	Cenpf
4.48E-49	2.1381	0.957	0.062	1.17E-44	5	CD4.sc6	Cdca8
2.30E-47	2.8141	1	0.08	6.02E-43	5	CD4.sc6	Pclaf
2.46E-45	2.1941	0.913	0.064	6.45E-41	5	CD4.sc6	Birc5
3.76E-43	2.3881	0.913	0.067	9.87E-39	5	CD4.sc6	Ube2c
1.27E-40	2.1334	1	0.098	3.34E-36	5	CD4.sc6	Hist1h4h
4.34E-40	2.2946	0.957	0.089	1.14E-35	5	CD4.sc6	Prc1
8.40E-38	2.1106	1	0.111	2.20E-33	5	CD4.sc6	Diaph3
1.16E-37	4.2177	1	0.122	3.05E-33	5	CD4.sc6	Hist1h1b
2.68E-35	2.3752	0.957	0.116	7.03E-31	5	CD4.sc6	Hist1h3e
4.67E-34	3.9954	1	0.142	1.22E-29	5	CD4.sc6	Hist1h2ae
5.19E-34	3.1412	1	0.138	1.36E-29	5	CD4.sc6	Mki67
6.56E-28	2.124	0.957	0.16	1.72E-23	5	CD4.sc6	Hist2h2ac
2.16E-27	3.3427	1	0.205	5.66E-23	5	CD4.sc6	Top2a
2.22E-26	2.0812	1	0.212	5.81E-22	5	CD4.sc6	Smc2
2.63E-25	1.9485	0.957	0.16	6.91E-21	5	CD4.sc6	Rad51b
1.17E-24	4.4356	1	0.247	3.06E-20	5	CD4.sc6	Hist1h2ap
1.38E-23	2.9391	1	0.26	3.62E-19	5	CD4.sc6	Stmn1
1.53E-19	2.034	1	0.365	4.01E-15	5	CD4.sc6	Lmnb1
8.97E-19	2.045	1	0.408	2.35E-14	5	CD4.sc6	Tuba1b
2.74E-18	3.2597	1	0.497	7.18E-14	5	CD4.sc6	Hist1h4d
8.10E-17	2.4069	1	0.675	2.13E-12	5	CD4.sc6	Tubb5
8.39E-16	3.1063	1	0.793	2.20E-11	5	CD4.sc6	Hmgb2
1.23E-15	2.6079	1	0.833	3.23E-11	5	CD4.sc6	H2afz
3.83E-15	2.2363	1	0.519	1.01E-10	5	CD4.sc6	Hist1h1e

Table S5: Genes associated to each MP

MP1	MP2	MP3	MP4
2010110K18Rik	4930533B01Rik	1700006J14Rik	0610005C13Rik
2810408I11Rik	9530026P05Rik	1700025G04Rik	4930405A10Rik
4930503L19Rik	AC149090.1	6030407O03Rik	4930453C13Rik
4930558J18Rik	Acacb	8030451A03Rik	9330175E14Rik
4930579G24Rik	Acvr1c	Abhd2	9930111J21Rik2
5430427M07Rik	Appl2	Abtb2	A530032D15Rik
Alms1	Bhlhe41	Acss3	A730085K08Rik
Anapc15	Bnc2	Adamts1	A930037H05Rik
Ankle1	C1ql3	Adamts12	AC168977.2
Anln	Cdh1	Adamts5	Aida
Anp32e	Cdk15	Adgrl2	Ankrd34a
Arhgap11a	Cdk2	Ahr	Apol6
Arhgap19	Cfap54	Aldh1a3	Apol9a
Arhgap33	Chsy3	Ano6	Apol9b
Arhgef39	Daam1	Antxr1	Atp8b4
Asf1b	Dennd5b	Aopep	Axl
Aspm	Disp1	Apobec2	B2m
Aspn	Ehbp1	Aqp1	Batf2
Atad2	Eps8	Arhgap10	Bst2
Atad5	Fmn1	Arhgap24	C1ra
Aunip	Gk	Arhgap29	Casp12
Aurka	Gm13963	Arih1	Ccl2
Aurkb	Gm2115	Arl15	Cfap100
BC030867	Lyst	Atp2b4	Cfb
BC055324	Man1a	Atxn1	Clec2d
Bard1	Man2a1	Baiap2l1	Cmpk2
Batf3	Met	Bcar3	Csf1
Baz1b	Mgat5	Bcas3	Csprs
Birc5	Nceh1	Bche	Csta2
Blm	Nedd4l	Bgn	Cxcl10
Bora	Nhs	C030034L19Rik	Cxcl9
Brca1	Oca2	Cacna1d	Ddx58
Brca2	Osbpl6	Cacnb3	Ddx60
Brd8	Padi2	Cadps	Dhx58
Brip1	Pde10a	Cblb	Dtx3l
Bub1	Ppargc1a	Cdk14	Eif2ak2
Bub1b	Ralgapa2	Cdk6	F830016B08Rik
C230066G23Rik	Sfi1	Col15a1	Flacc1
Camk2n2	Slc24a4	Col28a1	Gbp10
Casp8ap2	Slc7a5	Col3a1	Gbp2
Cbx5	St3gal6	Col5a2	Gbp3
Ccdc18	Sytl2	Col6a1	Gbp4
Ccdc34	Tex2	Col6a2	Gbp5
Ccdc77	Tmod1	Col8a1	Gbp6
Cchcr1	Tspan5	Cped1	Gbp7
Ccna2	Tyr	Cspg4	Gbp8

Ccnb1	Tyrp1	Ctnnal1	Gbp9
Ccnb2	Ubl3	Dact1	Gm10827
Ccne1		Diaph2	Gm11747
Ccne2		Dip2c	Gm12185
Ccnf		Dkk2	Gm13822
Ccsap		Dmd	Gm15893
Cdc20		Dock10	Gm19684
Cdc25b		Dst	Gm20559
Cdc25c		Eefsec	Gm26789
Cdc45		Efna5	Gm4070
Cdc6		Efnb1	Gm42517
Cdc7		Egflam	Gm43727
Cdca2		Eln	Gm44091
Cdca3		Etv5	Gm44148
Cdca5		Ext1	Gm4841
Cdca7		Fam110b	Gm4951
Cdca7l		Fam178b	Gm50237
Cdca8		Farp1	Gm5148
Cdk1		Fgfr1	Gm7030
Cdkn2d		Fign	Gm8773
Cdkn3		Fkbp5	Gpr149
Cdt1		Frmd6	Gpr65
Cenpa		Gli3	Gvin1
Cenpc1		Gm13919	H2-D1
Cenpe		Gm20754	H2-K1
Cenpf		Gm35188	H2-Q10
Cenph		Gm4117	H2-Q4
Cenpi		Gnaq	H2-T22
Cenpk		Hapln1	H2-T23
Cenpl		Hdac9	Herc6
Cenpm		Hivep2	Ifi203
Cenpn		Hmga2	Ifi204
Cenpp		Hs2st1	Ifi205
Cenpq		Hspb2	Ifi206
Cenps		Htr2a	Ifi207
Cenpu		Ica1	Ifi211
Cenpw		Igfbp4	Ifi27
Cep128		Il1rap	Ifi35
Cep192		Insr	Ifi44
Cep295		Itgbl1	Ifi47
Cep55		Itprid2	Ifih1
Cep57l1		Jmjd1c	Ifit1
Cep72		Kank4	Ifit2
Cep89		Kcnn2	Ifit3
Cfap97d2		Kif13a	Ifit3b
Cgas		Kif26b	Ifitm3
Chaf1a		Klf4	Igtp
Chaf1b		L1cam	Iigp1
Chek1		Lama4	Irf1

Chek2		Large1	Irf7
Chgb		Ldlrad3	Irgm1
Chtf18		Lgr4	Irgm2
Cip2a		Limch1	Isg15
Cit		Lmcd1	Isg20
Ckap2		Lpar3	Ky
Ckap2l		Lrch1	Lgals9
Ckap5		Lrch3	Lrfn1
Cks1b		Lrp1	Ly6e
Cks2		Maml2	Map3k19
Clspn		Map2	Mlkl
Cmc2		Map4	Mndal
Cnih2		Map4k3	Nlrc5
Cntrob		Mcam	Nmi
D430018E03Rik		Megf10	Oas1a
Dbf4		Mgp	Oas1g
Dcdc2a		Mir100hg	Oas2
Dck		Mllt3	Oas3
Dctpp1		Moxd1	Oasl1
Ddias		Mpz	Oasl2
Ddx11		Myo5b	Olfr56
Dek		Myof	Parp10
Depdc1a		Nav3	Parp11
Depdc1b		Ndst3	Parp12
Dhfr		Neat1	Parp14
Diaph3		Nebl	Parp9
Dlgap5		Nes	Phf11a
Dna2		Ngfr	Phf11b
Dnaaf2		Nhsl1	Phf11d
Dnajc9		Nxn	Pml
Dnmt1		P3h2	Psmb10
Dnph1		Pam	Psmb8
Dscc1		Pcdh9	Psmb9
Dsn1		Pcgf5	Psme1
Dtl		Pcsk6	Psme2
Dtymk		Pdgfb	Rnf19b
Dut		Pdgfra	Rnf213
Dynlt1a		Pdgfrb	Rsad2
Dynlt1f		Pdgfrl	Rtp4
E2f1		Pdlim4	Samd9l
E2f2		Pdlim5	Samhd1
E2f7		Pdzrn3	Slc2a6
E2f8		Pitpnc1	Slfn8
Ect2		Plcb1	Slfn9
Efcab11		Plekha6	Socs1
Eldr		Plpp1	Sp100
Eme1		Ppp1r1c	Sp110
Emp1		Prelp	Sp140
Ercc6l		Ptpn13	Stat1

Eri1		Ptprj	Stat2
Eri2		Rab27b	Styk1
Esco2		Ramp3	Tap1
Espl1		Rapgef2	Tap2
Exo1		Raph1	Tapbp
Exosc8		Rasgrp2	Tapbpl
Ezh2		Rbms1	Tgtp1
Fam111a		Rere	Tgtp2
Fam83d		Rgs16	Tnfsf10
Fanca		Rnd3	Tomm6os
Fancb		Robo1	Tor3a
Fancd2		Robo2	Trafd1
Fanci		Rock2	Trim12a
Fbxo48		Ror1	Trim12c
Fbxo5		S100a6	Trim21
Fen1		Samd4	Trim25
Fignl1		Samd5	Trim30a
Fkbp2		Sdc3	Trim34a
Fn3k		Sec14l5	Ube2l6
Foxg1		Sema3a	Usp18
Foxm1		Sema3b	Wnt2b
Fzr1		Sema5a	Xaf1
G2e3		Sema6a	Xdh
Gabrb3		Serpinf1	Ypel4
Gas2l3		Serping1	Zbp1
Gemin6		Sertad4	Znfx1
Gen1		Sh3rf1	
Gins1		Sipa1l1	
Gins2		Sipa1l3	
Gins3		Slc35f1	
Gins4		Slc9a3r2	
Gm14091		Sntb1	
Gm20628		Sorcs2	
Gm20667		Taf4b	
Gm3550		Tanc1	
Gm38037		Tbck	
Gm41555		Tcf20	
Gm42031		Tcim	
Gm42047		Tead1	
Gm44238		Tecrl	
Gm47207		Tenm3	
Gm4737		Tfap2b	
Gm48646		Thbs2	
Gm49014		Thsd4	
Gm550		Tiam2	
Gmnn		Tln2	
Gpsm2		Tnc	
Grm1		Trio	
Gtse1		Tslp	

H2afx		Ttc39b	
H2afz		Ube2e2	
Haspin		Wnt4	
Hat1		Wnt6	
Haus4		Xirp1	
Haus6		Zbtb38	
Haus8		Zeb1	
Hdgf		Zfc3h1	
Hells		Zfp532	
Hid1			
Hirip3			
Hist1h1a			
Hist1h1b			
Hist1h1c			
Hist1h1d			
Hist1h1e			
Hist1h1t			
Hist1h2ab			
Hist1h2ac			
Hist1h2ad			
Hist1h2ae			
Hist1h2af			
Hist1h2ag			
Hist1h2ah			
Hist1h2ai			
Hist1h2ak			
Hist1h2an			
Hist1h2ap			
Hist1h2bb			
Hist1h2be			
Hist1h2bf			
Hist1h2bg			
Hist1h2bh			
Hist1h2bj			
Hist1h2bk			
Hist1h2bl			
Hist1h2bm			
Hist1h2bn			
Hist1h2bp			
Hist1h2br			
Hist1h3a			
Hist1h3b			
Hist1h3c			
Hist1h3e			
Hist1h3f			
Hist1h3g			
Hist1h3h			
Hist1h3i			
Hist1h4a			

Hist1h4b			
Hist1h4c			
Hist1h4d			
Hist1h4f			
Hist1h4h			
Hist1h4j			
Hist1h4k			
Hist1h4n			
Hist2h2aa1			
Hist2h2ab			
Hist2h2ac			
Hist2h2bb			
Hist2h3b			
Hjurp			
Hmgb2			
Hmgb3			
Hmgn2			
Hmgn5			
Hmgxb4			
Hmmr			
Hmx2			
Hyls1			
Il1rapl2			
Impa2			
Incenp			
Iqgap3			
Jpt1			
Kctd18			
Kif11			
Kif14			
Kif15			
Kif18a			
Kif18b			
Kif20a			
Kif20b			
Kif22			
Kif23			
Kif24			
Kif2c			
Kif4			
Kifc1			
Kifc5b			
Knl1			
Knstrn			
Kntc1			
Kpna2			
Lbr			
Lig1			
Lin54			

Lin9			
Lmnb1			
Lockd			
Lrfn1			
Lrr1			
Lsm2			
Lsm5			
Mad2l1			
Mastl			
Mcm10			
Mcm2			
Mcm3			
Mcm4			
Mcm5			
Mcm6			
Mcm7			
Mdc1			
Melk			
Mis18a			
Mis18bp1			
Mki67			
Mlf1			
Mms22l			
Mnd1			
Mns1			
Mtbp			
Mtfr2			
Mthfd2			
Mxd3			
Myb			
Mybl1			
Mybl2			
Nasp			
Ncapd2			
Ncapd3			
Ncapg			
Ncapg2			
Ncaph			
Ndc1			
Ndc80			
Nde1			
Neil3			
Nek2			
Nell1			
Nemp1			
Neurl1b			
Nfatc2ip			
Nmral1			
Nrm			

Nsd2			
Nsl1			
Nt5dc2			
Nucks1			
Nuf2			
Nup107			
Nup155			
Nup205			
Nup35			
Nup37			
Nup43			
Nup85			
Nusap1			
Nxt1			
Odf2			
Ogn			
Oip5			
Omd			
Orc6			
Otx2			
Parpbp			
Pask			
Pbk			
Pclaf			
Pcna			
Phf19			
Pif1			
Pimreg			
Pkmyt1			
Pla2g4c			
Plk1			
Plk4			
Pmf1			
Pola1			
Pola2			
Pold1			
Pold2			
Pold3			
Pole			
Pole2			
Polh			
Polq			
Prc1			
Prim1			
Prim2			
Prr11			
Psmc3ip			
Racgap1			
Rad18			

Rad21			
Rad50			
Rad51			
Rad51ap1			
Rad51b			
Rad54b			
Rad54l			
Rangap1			
Rbbp8			
Rbl1			
Rccd1			
Reep4			
Rfc1			
Rfc2			
Rfc3			
Rfc4			
Rfc5			
Rfwd3			
Rif1			
Rmi2			
Rnaseh2c			
Rnf168			
Rpa2			
Rpa3			
Rrm1			
Rrm2			
Rtkn2			
Sapcd2			
Sass6			
Scml2			
Selenoh			
Serpinb8			
Sgo1			
Sgo2a			
Shcbp1			
Siva1			
Ska1			
Ska2			
Ska3			
Skp2			
Slbp			
Slc30a10			
Slc43a3			
Slc9a5			
Slf1			
Slfn9			
Smc2			
Smc4			
Snrnp25			

Spag5			
Spc24			
Spc25			
Spdl1			
Stbd1			
Stil			
Stmn1			
Suv39h1			
Tacc3			
Tagln2			
Tbc1d31			
Tcf19			
Tedc1			
Terf1			
Tex30			
Tfdp1			
Ticrr			
Timeless			
Tipin			
Tk1			
Tmpo			
Tnfaip8l1			
Top2a			
Topbp1			
Tpx2			
Traip			
Trim59			
Trip13			
Troap			
Ttk			
Tuba1c			
Tubb4b			
Tubb6			
Tube1			
Tubg1			
Tyms			
Ube2c			
Ube2s			
Ube2t			
Uevld			
Uhrf1			
Ulbp1			
Ung			
Usp1			
Usp37			
Vrk1			
Wdhd1			
Wdr76			
Wee1			

Xkr5			
Xndc1			
Zfand4			
Zfp367			
Zfp993			
Zgrf1			
Zranb3			
Zwilch			

MP5	MP6	MP7
1110008P14Rik	1700016K05Rik	Acaca
2310009B15Rik	1700025G04Rik	Acacb
Abracl	2610035D17Rik	Aph1c
Ak6	4930517O19Rik	Bri3
Akr1a1	4930554G24Rik	Chpt1
Akr1b3	Adam12	Cited1
Anp32b	Adamts5	Cox17
Anp32e	Adamts6	Cox8b
Anxa2	Adgrl3	Cryab
Ap2m1	Aff3	Cst6
Ap2s1	Agap1	Cyba
Apex1	Akap12	Cystm1
Aprt	Arap2	Dct
Arf1	Asap1	Eif3f
Arhgdib	Atrnl1	Fabp3
Arpc3	B4galt6	Fabp5
Arpp19	Cd200	Fam104a
Asna1	Cd9	Foxp2
Atp5c1	Cdh10	Fundc2
Atp5d	Cdk5rap2	Gm3776
Atp5f1	Chl1	Gng8
Atp5g1	Col23a1	Gsk3b
Atp5h	Csad	Gsta1
Atp5j	Ctsh	Gsta2
Atp5k	Cttnbp2	Gstp1
Atp5o	Dkk3	Mlana
Atp6v0e	Dock3	Mtss1
Atpif1	Eno3	Myo5a
Aurkaip1	Erbb3	Ndufs2
Bag1	Etv1	Oat
Banf1	Fam184b	Oca2
Bax	Fap	Pmel
Bex3	Fbxl7	Ptgds
Bola2	Fxyd1	Ptprs
Btf3	Gjc3	Ralgapa2
Bud31	Gm15594	Reep5
Bzw1	Gm49267	Rgs10
C1qbp	Gpm6b	Rhoa
Cacybp	Gulp1	Rnasek
Ccdc124	Hecw2	Rnf7
Cct3	Hpgds	S100a1
Cct5	Ikbkb	S100b
Cct6a	Il16	Sec11c
Cct8	Inpp5f	Selenos
Cdk2ap1	Iqgap2	Sgcd
Cdk4	Itga2	Slc37a2

Cenpx	Klf12	Smpd1
Cetn3	Lgi4	Traf5
Chchd1	Lima1	Tspan10
Chchd4	Lmo4	Tyrp1
Chchd7	Map4k3	Uqcrfs1
Chmp2a	Mbp	Vegfb
Churc1	Mecom	Vps29
Ciao2b	Mef2c	
Cisd1	Megf9	
Clic1	Metrn	
Cnbp	Mmd	
Cnih4	Mprip	
Coa3	Mpzl1	
Commd1	Mtss1	
Cope	Mxra8	
Cops6	Myzap	
Cops9	Ndrg2	
Coq7	Nrn1	
Cox20	Ntn4	
Cox5a	P3h2	
Cox5b	Pde1c	
Cox6a1	Plat	
Cox7b	Plpp1	
Creld2	Ppm1l	
Cryab	Prickle2	
Cstb	Prkca	
Cuedc2	Prss23	
Cyc1	Psd3	
Cycs	Ptprm	
Dctn3	Rasal2	
Dctpp1	Rasgrp3	
Ddt	Rbfox2	
Ddx21	Rbm20	
Ddx39	Rnf152	
Denr	Sema3d	
Dynll1	Serpinb1a	
Ebna1bp2	Sgcd	
Eef1d	Slc12a2	
Eef1e1	Slc4a8	
Eif1ad	Snx25	
Eif2s1	Sort1	
Eif2s2	Sox5	
Eif3b	Sox5os4	
Eif3c	Sox6	
Eif3g	Stard13	
Eif3i	Tcf7l1	
Eif3j1	Tgfb2	
Eif4a1	Tnik	
Eif4e2	Tom1l2	

Eif4g2	Unc5c	
Eif5a	Zbtb7c	
Eif6	Zfhx4	
Elob	Zfp608	
Elof1	Zfp704	
Emc6		
Emg1		
Eno1		
Erh		
Esd		
Etfb		
Fabp5		
Fam162a		
Fbl		
Fkbp11		
Fkbp1a		
Fkbp3		
Fkbp4		
G3bp1		
Gadd45gip1		
Galk1		
Gapdh		
Gemin6		
Glrx3		
Gm2000		
Gnai2		
Gnb2		
Gnl3		
Gpx1		
Gspt1		
Gtf2h5		
Higd1a		
Hint1		
Hmgn1		
Hnrnpa3		
Hnrnpab		
Hnrnpd		
Hnrnpf		
Hras		
Hsp90aa1		
Hspa5		
Hspa8		
Hspd1		
Hspe1		
Idh2		
Idh3a		
Ier3ip1		
Ifitm2		
Impdh2		

Jpt1		
Kmt5a		
Lamtor1		
Lamtor4		
Lamtor5		
Ldha		
Llph		
Lrrc59		
Lsm12		
Lsm2		
Lsm4		
Lyar		
Magoh		
Mak16		
Manf		
Mbd3		
Mdh1		
Metap2		
Micos10		
Micos13		
Mien1		
Morf4l1		
Mpc2		
Mrpl12		
Mrpl13		
Mrpl14		
Mrpl17		
Mrpl18		
Mrpl20		
Mrpl21		
Mrpl23		
Mrpl27		
Mrpl28		
Mrpl30		
Mrpl34		
Mrpl35		
Mrpl36		
Mrpl52		
Mrpl53		
Mrpl54		
Mrpl57		
Mrpl58		
Mrps14		
Mrps16		
Mrps21		
Mrps24		
Mrps36		
Mrps7		
Mrto4		

Mvb12a		
Mybbp1a		
Mydgf		
Myl12a		
Myl6		
Naa38		
Nap1l1		
Ncl		
Ndufa10		
Ndufa11		
Ndufa12		
Ndufa3		
Ndufa5		
Ndufa8		
Ndufab1		
Ndufb2		
Ndufb3		
Ndufb6		
Ndufb7		
Ndufb8		
Ndufs3		
Ndufs5		
Ndufs6		
Ndufs7		
Ndufs8		
Ndufv2		
Ndufv3		
Nhp2		
Nifk		
Nme1		
Nol7		
Nolc1		
Nop10		
Nop56		
Nop58		
Npm3		
Nsa2		
Nudc		
Nudcd2		
Oaz1		
Pa2g4		
Pabpc1		
Park7		
Pcbp1		
Pdap1		
Pdcd5		
Pebp1		
Pet100		
Pfdn2		

Pfdn6		
Pfn1		
Pgam1		
Pgk1		
Pgls		
Phb2		
Phf5a		
Pin1		
Pkm		
Pole3		
Polr1d		
Polr2e		
Polr2f		
Polr2g		
Polr2j		
Polr2k		
Polr2l		
Pomp		
Pop5		
Pop7		
Ppa1		
Ppdpf		
Ppp1ca		
Ppp1r14b		
Prdx6		
Prelid1		
Prelid3b		
Prkar1a		
Prmt1		
Psenen		
Psma2		
Psma3		
Psma4		
Psma5		
Psma6		
Psma7		
Psmb1		
Psmb2		
Psmb3		
Psmb4		
Psmb5		
Psmb6		
Psmc2		
Psmc3		
Psmc4		
Psmc5		
Psmd12		
Psmd6		
Psmd7		

Psmd8		
Psmg4		
Ptges3		
Rab5if		
Rad23a		
Ran		
Ranbp1		
Rbis		
Rbm8a		
Rer1		
Rhoa		
Rhoc		
Rpl12		
Rpl23a		
Rpl31		
Rps17		
Rps19bp1		
Rps25		
Rps26		
Rsl1d1		
Ruvbl1		
Rwdd1		
S100a10		
Sap18		
Sdhb		
Sdhd		
Serbp1		
Set		
Sf3b5		
Sf3b6		
Sh3bgrl3		
Skp1a		
Slc25a39		
Slc25a5		
Slirp		
Smarca5		
Snrnp27		
Snrpb		
Snrpc		
Snrpd1		
Snrpd2		
Snrpd3		
Snrpf		
Snrpg		
Snu13		
Snx3		
Sparc		
Spcs1		
Sra1		

Srm		
Srp19		
Srsf3		
Ssb		
Ssr4		
Strap		
Sub1		
Suclg1		
Sumo2		
Svbp		
Swi5		
Taf10		
Taldo1		
Tbcb		
Timm10		
Timm10b		
Timm13		
Timm23		
Timm8a1		
Tmed9		
Tmem126a		
Tmem14c		
Tmem208		
Tmem258		
Tomm22		
Tomm5		
Tpi1		
Tpm3		
Tpm4		
Trappc2l		
Trir		
Trmt112		
Tspo		
Txn2		
Txndc17		
Txnl1		
Txnl4a		
U2af1		
Ube2m		
Ufc1		
Uqcc2		
Uqcr10		
Uqcrb		
Vdac1		
Vdac2		
Vdac3		
Wdr83os		
Xbp1		
Ybx3		

Ywhab		
Ywhaq		
Ywhaz		
Zc3h15		
Zmat2		
Znhit1		

Table S6: Patient T cell, BR1 and BR3 genes signatures

Wischnewski_2023 (BrM pTRT CD8)	VanderLeun_2020	Caushi_2021	Lowery_2022	Hanada_2022	Oliviera_2021
CXCL13	CXCL13	ZNF683	ATP10D	ENTPD1	KRT86
RGS1	ENTPD1	GEM	GZMB	CXCL13	RDH10
CCL4L2	ITGAE	TOX2	ENTPD1	HMOX1	TYMS
GZMB	PDCD1	BATF	KIR2DL4	PDCD1	HMOX1
DUSP4	HAVCR2	GBP5	LAYN	LAYN	GNG4
IFNG	LAG3	MIR4435-2HG	HTRA1	CD27	CXCL13
TRBV28	TNFRSF18	TNS3	CD70	HAVCR2	AFAP1L2
GAPDH	TNFRSF9	GNA15	CXCR6	TNFRSF9	ACP5
CTLA4		CXCL13	HMOX1	MIR155HG	MYO1E
CCL3		RBPJ	ADGRG1	BATF	LAYN
LAG3		ENTPD1	LRRN3	TIGIT	TNS3
CD7		LINC02195	ACP5	AD000671.2	TNFSF4
TIGIT		GPR25	CTSW	GZMH	AKAP5
CCL4		ITGAE	GALNT2	CD70	HAVCR2
ITGAE		GNLY	LINC01480	TMEM121	ENTPD1
		PDCD1	CARS	LRRN3	SLC2A8
		CTLA4	LAG3	NHS	AC243829.4
		PRDM1	TOX	TTN	ZBED2
		TNFRSF9	PTPRCAP	LINC01281	MCM5
		TIGIT	ASB2	ASB2	CAV1
		HAVCR2	ITGB7	SIRPG	GOLIM4
			PTMS	ANKS1B	TRAV21
			CD8A		VCAM1
			GPR68		PON2
			NSMCE1		MTSS1
			ABI3		CD38
			SLC1A4		TRBV11-2
			PLEKHF1		MS4A6A
			CD8B		TOX2
			LINC01871		CSF1
			CCL4		GALNT2
			NKG7		FXYD2
			CLIC3		PLPP1
			NDFIP2		LMCD1
			PLPP1		MYL6B
			PCED1B		LAG3
			CXCL13		HLA-DRA
			PDCD1		IGFLR1

			PRF1		CCDC50
			HLA-DMA		CD27
					KIAA1324
					CDKN2A
					CD70
					ABHD6
					CTLA4
					PDCD1
					GEM
					NUSAP1
					TOX
					CXCR6
					NMB
					HOPX
					CLIC3
					INPP5F
					SNAP47
					TSHZ2
					HLA-DMA
					SIT1
					HLA-DRB1
					TUBB
					PYCARD
					ADGRG1
					HLA-DQA1
					PRF1
					HLA-DPA1
					PTMS
					CKS1B
					HIPK2
					CHST12
					LSP1
					FAM3C
					SLC1A4
					NUDT1
					DNPH1

MP4	MP6	BR1 pro-inflammatory	T_CD8	BR1 chemokine L-R	BR3 Neuro
AIDA	C1orf21	HLA-DRB5	CD8A	CCL3	SEMA3A
ANKRD34A	ADAM12	HLA-DQA1	CD8B	CCL4	SEMA6C
APOL6	ADAMTS5	CD74	CD3E	CCL5	SEMA6B
APOL1	ADAMTS6	TAP1		CCR5	SEMA6A
APOL2	ADGRL3	HLA-DQB2		CXCR6	
APOL1	AFF3	HLA-DQB1		CXCR3	
APOL2	AGAP1	HLA-E		CXCL16	
ATP8B4	AGAP11	B2M		CXCL9	
AXL	AGAP5	CCL5			
B2M	AGAP7P	CCL7			
BATF2	AGAP9	CCL2			
BST2	AKAP12	CXCL10			
C1R	ARAP2	CXCL16			
CASP12	ASAP1	CXCL9			
CCL13	ATRNL1	CCL1			
CCL2	B4GALT6	ISG15			
CFAP100	CD200	IFIT3			
CFB	CD9	IFIT2			
CLEC2D	CDH10	IFI35			
CMPK2	CDK5RAP2	IFI27L2			
CSF1	CHL1	SPP1			
CSTA	COL23A1	IFNB1			
CXCL10	CSAD	TNF			
CXCL9	CTSH	S100A6			
DDX60	CTTNBP2	S100A4			
DHX58	DKK3	ITGAX			
DTX3L	DOCK3	LGALS1			
EIF2AK2	ENO3	LGALS3			
FLACC1	ERBB3	CD86			
GBP6	ETV1	MKI67			
GBP1	FAM184B	CENPF			
GBP2	FAP	BIRC5			
GBP4	FBXL7	TOP2A			
GBP7	FXYD1	CCNB2			
GBP6	GJC3				
GBP5	GPM6B				
GBP6	GULP1				
GBP7	HECW2				

GBP6	HPGDS				
GBP6	IKBKB				
GPR149	IL16				
GPR65	INPP5F				
GVINP1	IQGAP2				
HLA-A	ITGA2				
HLA-B	KLF12				
HLA-C	LGI4				
HLA-E	LIMA1				
HLA-F	LMO4				
HLA-G	MAP4K3				
HLA-H	MBP				
HLA-A	MECOM				
HLA-B	MEF2C				
HLA-C	MEGF9				
HLA-E	METRN				
HLA-F	MMD				
HLA-G	MPRIP				
HLA-A	MPZL1				
HLA-B	MTSS1				
HLA-C	MXRA8				
HLA-E	GCOM1				
HLA-F	MYZAP				
HLA-G	POLR2M				
HLA-A	NDRG2				
HLA-B	NRN1				
HLA-C	NTN4				
HLA-E	P3H2				
HLA-B	PDE1C				
HLA-C	PLAT				
HLA-E	PLPP1				
HLA-F	PPM1L				
HLA-G	PRICKLE2				
HLA-E	PRKCA				
HERC6	PRSS23				
IFI16	PSD3				
IFI16	PTPRM				
MNDA	RASAL2				
PYHIN1	RASGRP3				
IFI16	RBFOX2				
MNDA	RBM20				
PYHIN1	RNF152				
IFI16	SEMA3D				

IFI16	SERPINB1				
IFI16	SGCD				
MNDA	SLC12A2				
PYHIN1	SLC4A8				
IFI27	SNX25				
IFI27L1	SORT1				
IFI27L2	SOX5				
IFI35	SOX6				
IFI44	STARD13				
IFIH1	TCF7L1				
IFIT1	TGFB2				
IFIT1B	TNIK				
IFIT2	TOM1L2				
IFIT3	UNC5C				
IFIT3	ZBTB7C				
IFITM3	ZFHX4				
IRGM	ZNF608				
IRF1	ZNF704				
IRF7	Zfp704				
IRGM					
IRGM					
ISG15					
ISG20					
KY					
LGALS9					
LGALS9B					
LGALS9C					
LRFN1					
LY6E					
MAP3K19					
MLKL					
IFI16					
NLRC5					
NMI					
OAS1					
OAS1					
OAS2					
OAS3					
OASL					
OASL2P					
PARP10					
PARP11					
PARP12					

PARP14					
PARP9					
PHF11					
PHF11					
PHF11					
PML					
PSMB10					
PSMB8					
PSMB9					
PSME1					
PSME2					
RNF19B					
RNF213					
RSAD2					
RTP4					
SAMD9L					
SAMHD1					
SLC2A6					
SLFN11					
SLFN13					
SLFN11					
SLFN13					
SOCS1					
SP100					
SP110					
SP140					
SP140L					
STAT1					
STAT2					
STYK1					
TAP1					
TAP2					
TAPBP					
TAPBPL					
TNFSF10					
TOR3A					
TRAFD1					
TRIM5					
TRIM5					
TRIM21					
TRIM25					
TRIM5					
TRIM34					

TRIM6-TRIM34					
UBE2L6					
USP18					
USP41P					
WNT2B					
XAF1					
XDH					
YPEL4					
ZBP1					
ZNFX1					
